# Elucidating the roles of essential genes in autotrophic metabolism and cell morphology of *Clostridium ljungdahlii* by CRISPRi

**DOI:** 10.1007/s00253-026-13714-3

**Published:** 2026-01-27

**Authors:** Saira Munir, Sai Wan, Xinyu Gao, Mingchi Lai, Zhenjie Mu, Hui Wang, Ziyong Liu, Fuli Li, Lin Xia, Yang Tan

**Affiliations:** 1 State Key Laboratory of Photoelectric Conversion and Utilization of Solar Energy, Shandong C1 Refinery Engineering Research Center, Qingdao New Energy Shandong Laboratory, Qingdao Institute of Bioenergy and Bioprocess Technology, Chinese Academy of Sciences, Qingdao, Shandong 266101 China; 2 Chinese Medicine Guangdong Laboratory (Hengqin Laboratory), Guangdong-Macao In-Depth Cooperation Zone in Hengqin, Guangdong, 519000 China; 3 University of Chinese Academy of Sciences, Beijing, China; 4 Shenzhen Institutes of Advanced Technology, Chinese Academy of Sciences, Shenzhen, China

**Keywords:** *Clostridium ljungdahlii*, Syngas fermentation, CRISPR interference, Autotrophic metabolism, Inducible promoters, Essential genes

## Abstract

Understanding the function of essential genes in *Clostridium ljungdahlii* is critical for unraveling its autotrophic metabolism and optimizing its potential as a platform for syngas fermentation. However, study on essential genes of this species remains insufficient. Here, we employed an inducible CRISPR interference (CRISPRi) system to investigate the roles of key metabolic and cell division genes in *C. ljungdahlii.* Targeted repression of genes encoding pyruvate:ferredoxin oxidoreductase (PFOR1, PFOR2), acetaldehyde:ferredoxin oxidoreductase (AOR1, AOR2), and glyceraldehyde phosphate hydrogenase type I (GAP-I) revealed their essential contributions to autotrophic growth, as knockdown strains exhibited impaired growth and reduced ethanol production. Furthermore, downregulation of the cell division gene *ftsZ* resulted in elongated cell morphology, highlighting its critical role in cell shape regulation. These findings provide new insights into the functional importance of essential genes in *C. ljungdahlii* and demonstrate how targeted gene repression can advance our understanding of autotrophic metabolism and cellular processes.

**Supplementary Information:**

The online version contains supplementary material available at 10.1007/s00253-026-13714-3.

## Introduction

Synthesis gas (CO_2_, CO, and H_2_) can be converted into biofuels and biochemicals, which have recently attracted much attention for reducing the emission of greenhouse gas (De Tissera et al. 2019; Liu et al. 2023). Some acetogenic bacteria have a natural capability to convert the syngas into organic compounds with two or more carbons such as acetate, ethanol, butyrate, butanol, or 2,3-butanediol (Pavan et al. 2022; Rosenbaum and Müller 2023). Of the acetogenic bacteria, *C. ljungdahlii* has the ability of converting syngas into ethanol with high efficiency (Klasson et al. 1993). Many studies have been conducted to elucidate the mechanism for one-carbon fixation, energy conservation, and product synthesis in *C. ljungdahlii* (Buckel and Thauer 2018; Herrmann et al. 2008; Köpke et al. 2010; Muller 2003; Schuchmann and Müller 2014). Moreover, *C. ljungdahlii* is genetically tractable and its genetic transformation is available (Leang et al. 2013). *C. ljungdahlii* has already become one well-known model microorganism and represented one important platform for the syngas fermentation (Zhang et al. 2020).

Functions of some genes in *C. ljungdahlii* have been elucidated preliminarily since the development of its genetic system (Huang et al. 2016), but major limitations remain when studying essential genes. Homologous recombination-based gene deletion, while useful for gene knockouts, is inefficient and time-consuming (Leang et al. 2013). CRISPR-Cas genome editing, though powerful, cannot be easily applied to essential genes because their complete deletion is lethal (Zhao et al. 2019). As a result, alternative strategies are needed to investigate the function of indispensable metabolic and cellular machinery without permanently disrupting them.

Alternatively, an enzymatically inactive variant of Cas protein, such as dCas9 or dCas12a, can be employed to target specific genomic loci and block transcription without cleaving DNA (Bikard et al. 2013; Gilbert et al. 2013). When combined with a single guide RNA (sgRNA), the dCas protein sterically hinders transcription at the sgRNA-complementary locus (Qi et al. 2013). This approach has been successfully implemented in various bacteria, including *C. ljungdahlii*, *Escherichia coli* (Qi et al. 2013), *Bacillus subtilis* (Peters et al. 2016), *Streptococcus pneumoniae* (Jana et al. 2024), and *Mycobacterium tuberculosis* (Li et al. 2022), facilitating robust genome-wide knockdown studies. In *C. ljungdahlii*, CRISPRi systems have been used to regulate genes with known functions, e.g., phosphotransacetylase (PTA), to modulate metabolic flux (Woolston et al. 2018; Zhao et al. 2019). However, the full potential of CRISPRi, particularly its utility in elucidating essential gene functions, is underexploited.

While key metabolic and cell division genes such as *pfor1*, *pfor2*, *aor1*,* aor2*,* gap1* and *gap2*, *ftsZ*, and *mreB* are well studied in other bacteria, their regulatory roles and physiological consequences of *C. ljungdahlii* remain poorly understood. This organism couples carbon fixation to energy conservation through a strictly anaerobic, redox-limited metabolism. These conditions may fundamentally alter how these conserved genes function and interact. Understanding their regulation and phenotypic effects in *C. ljungdahlii* is therefore critical for uncovering acetogen-specified mechanisms that cannot be inferred from model heterotrophs.

In this study, we leveraged an inducible CRISPRi system to dissect the roles of critical genes in *C. ljungdahlii*. We repressed the expression of key enzymes in the Wood-Ljungdahl pathway, central metabolism and cell division machinery. Our goal was to answer this question: how does down-regulation of these essential genes affect autotrophic growth, energy metabolism, and cell morphology in an acetogen? Our findings not only advance the understanding of core metabolic functions in *C. ljungdahlii*, but also improve a framework for future studies on gene function in this industrially relevant microorganism.

## Materials and methods

### Bacterial strains and growth conditions

Molecular cloning experiments were performed using *E. coli* DH5α, which were cultured in Luria-Bertani (LB) medium, and 500 µg/ml erythromycin was used when needed. The *C. ljungdahlii* DSM 13528 and its derived strains were anaerobically cultured in the yeast extract-tryptone-fructose (YTF) medium (Humphreys et al. 2015) for regular strain maintenance. The modified PETC medium (Leang et al. 2013) with 5 g/l of fructose as the sole carbon (named as PETC[Fructose]) was used for heterotrophic growth, while the PETC medium with a headspace of 80% CO and 20% CO_2_ (named as PETC[CO/CO_2_]) or 25% CO_2_ and 75% H_2_ (named as PETC[CO_2_/H_2_]) pressurized to 0.2 Mpa as the sole carbon source was used for autotrophic fermentation. All of the manipulations for the *C. ljungdahlii* DSM 13528 were performed in a COY anaerobic chamber (Grass Lake, MI, USA) under 37 °C. Clarithromycin (5 µg/ml) was added into the PETC medium when needed.

### Plasmid construction

**Fig. 1:**
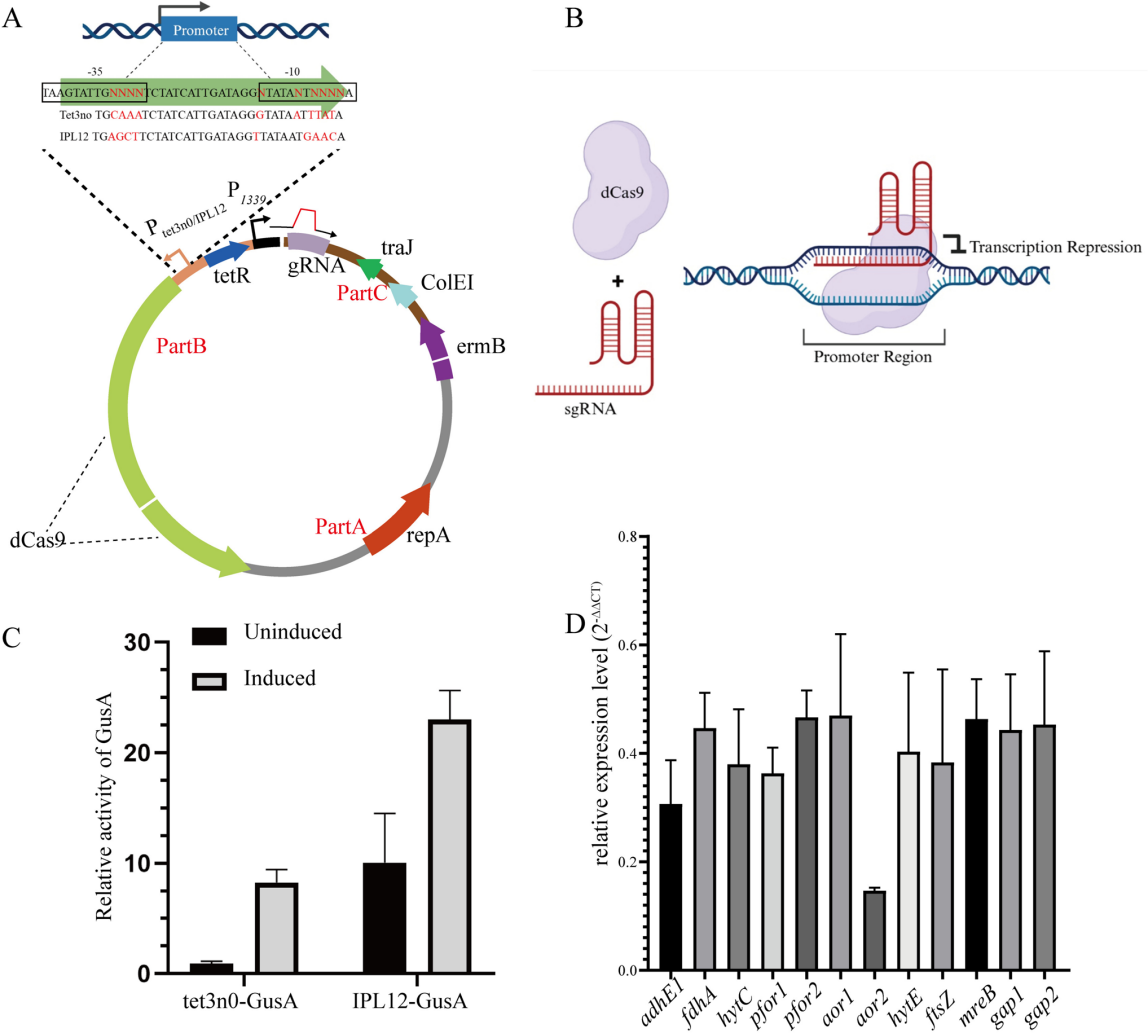
**A** The synthesized inducible promoters (Tet3n0 and IPL12) and the inducible CRISPRi vectors resulting from a three-fragment ligation approach. **B** Schematic illustration of the CRISPRi system, in which dCas9 together with sgRNA forms the dCas9/sgRNA complex and targets the gene sequence with an adjacent PAM where it can repress the transcription. **C** Strength of Tet3n0 and IPL12 promoter, which is represented by the relative activity of GusA. The activity is normalized to the activity of GusA under the uninduced Tet3n0 promoter. **D** Relative expression levels of target essential genes (*adhE1*, *fdhA*, *hytC*, *pfor1*, *pfor2*, *aor1*, *aor2*, *hytE*, *ftsZ*, *merB*, *gap1*, and *gap2*) after aTc-induced CRISPRi, which are revealed by qRT-PCR. All data above are representative of three replicates

For the inducible CRISPRi system, a nuclease-deficient Cas9 (dCas9) was obtained by generating two key amino acid residue mutations (D10A, H840A) (Qi et al. 2013) of Cas9 from the plasmid pMTLcas-pta (Huang et al. 2016). Then, the *dCas9* gene was assembled with IPL12/Tet3n0 promoter, a TetR repressor expression cassette, a sgRNA expression cassette controlled by P_*araE*_ (P_*1339*_) promoter, and linear plasmid pMTL82254 (digested with NotI/XhoI) used as backbone. In addition, the 20-bp protospacers for target genes were designed with the GuideMaker v0.4.2 software (Poudel et al. 2022). Two gRNAs were designed for each gene and one gRNA was selected with the most effective knockdown phenotype. gRNAs and the targeted gene IDs and gene names are listed in Table S2. The schematic diagram for the construction of CRISPRi plasmid is displayed in Fig. 1. The target genes were *hytC* and *hytE1* (encoding hydrogenase), *fdhA* (encoding formate dehydrogenase), *pfor1* and *pfor2* (encoding pyruvate:ferredoxin oxidoreductase), *adhE1* (encoding aldehyde/alcohol dehydrogenase), *aor1* and *aor2* (encoding acetaldehyde:ferredoxin oxidoreductase), *ftsz* (encoding filamenting temperature-sensitive mutant Z), and *mreB* (encoding an actin homologue). Gene ID and corresponding CRISPRi plasmids are listed in Table S1.

Furthermore, in order to facilitate the construction of these plasmids, we designed one three-fragment ligation system (Fig. 1). This system consists of fragment A (containing repA and a portion of dCas9), fragment B (containing the P1339 promoter, either the IPL12 or Tet3n0 promoter, and part of dCas9), and fragment C (containing the sgRNA scaffold, traj, ColE1, and ermB). In the PCR process, CRISPRi plasmids with IPL12 or Tet3n0 promoter were used as templates respectively. The fragments A and B were amplified using the fragment-A-F/R and fragment-B-F/R primers, respectively. For the amplification of fragment C, a 76-nt forward primer (fragment-C-F, containing a 20-nt spacer) was designed. A mixture of 6~10 forward primers with different spacers and a common reverse primer fragment-C-R were used to generate products for ligation. Thus, 6~10 CRISPRi plasmids can be constructed in one experiment. Additionally, we constructed control plasmids expressing dCas9 under the IPL12 or Tet3n0 promoter, but devoid of the sgRNA expression cassette. These plasmids allowed the evaluation of whether dCas9 expression alone represses target gene transcription. Further control plasmids included those expressing only the gRNA or the non-targeting RNA in combination with dCas9, to determine whether off-target gRNAs could mediate repression, both in the presence and absence of aTc induction.

All fragments were amplified with a Phanta Max Super-Fidelity DNA Polymerase (Vazyme Biotech Co., Ltd., Nanjing, China), and subjected to 1% w/v agarose gel electrophoresis and purified with an E.Z.N.A Gel extraction kit. All primers for the PCR are listed in Table S2. The DNA fragments are assembled by the ClonExpress II One Step Cloning Kit (Vazyme Biotech Co., Ltd., Nanjing, China) according to the manufacturer’s introduction. The DNA assembly product was transformed into the *E. coli* strains; the positive colony was identified through colony PCR. After the plasmids were extracted from the positive colony, Sanger sequencing was performed to confirm the sequence.

### Electrocompetent cell preparation and Electrotransformation of *C. ljungdahlii*

Electrocompetent cells were prepared following the protocol described before (Zhao et al. 2019) with minor modifications. Cells of *C. ljungdahlii* stored in −80 ℃ were transferred into 10-ml YTF medium and subcultured twice at 37 ℃. Then, the activity recovered cells were inoculated into 300-ml YTF medium with an initial OD_600nm_ of ~0.05 for overnight growth. When the cell OD_600nm_ reached 0.3–0.5, sucrose and glycine were added into the bottle to final concentrations of 0.3 M and 0.17 M, respectively, for 2-h incubation. The treated cells were collected by centrifugation for 10 min at 4000×g and 4 ℃, and then washed three times with 50-ml pre-chilled SMP buffer. At last, the washed cells were resuspended in 600 µl 10% DMSO dissolved in SMP buffer and stored at −80 ℃.

The electroporation was performed as previously reported (Leang et al. 2013) with minor changes. Briefly, 50-µl electrocompetent *C. ljungdahlii* cells stored at −80 ℃ were thawed on ice and mixed with 1 µg (less than 5 µl) plasmid DNA. The cells were transferred into a pre-chilled 1-mm-gap electroporation cuvette (Bio-Rad) and then electroporated at 0.625 kV, 600 Ω, and 25 µF with a BTX ECM 360, immediately after which the cells were transferred to 10-ml YTF at 37 ℃ for about 6~9-h incubation. The incubated 10-ml cultures were mixed with 20-ml YTF supplemented with clarithromycin (5 µg/ml) and allowed for growing for another 3~7 days, followed by plate spreading and single colony picking. All consumable materials mentioned above were placed in the anaerobic chamber overnight to remove oxygen, and the manipulations were carried out under anaerobic conditions. Finally, colony PCR was performed to obtain the target strains.

### Glucuronidase activity and promoter strength assay

To evaluate the strengths of inducible promoter IPL12 and Tet3n0, beta-glucuronidase gene (*gusA*) (Zhang et al. 2022) was synthesized and ligated with linear pMTL82254, expression of which was controlled by IPL12 or Tet3n0, respectively. The transformants carrying *gusA* expression plasmid (pIPL12/Tet3n0-GusA) were obtained as described by Mordaka and Heap (2018) with minor modifications.

For the assay of promoter strength, in brief, the strains harboring pIPL12/Tet3n0-GusA were cultured in YTF medium with an initial OD_600nm_ of 0.05 anaerobically at 37 ℃ overnight. When the OD_600nm_ reached 0.5, 35 ng/ml of anhydrotetracycline hydrochloride (aTc) was added to both the experimental group cultures and the control group, and each group includes three replicates. After 6 h from the induction, 2 ml of the cultures was pelleted and frozen at −80 ℃. For testing, cell pellets were resuspended in buffer (60 mM Na_2_HPO_4_·H_2_O, 40 mM NaH_2_PO_4_·H_2_O, 10 mM KCl, 1 mM MgSO_4_·7H_2_O, pH adjusted to 7.0, and 50 mM β-mercaptoethanol added freshly), and lysed by sonication on ice. Then, supernatants were collected and used for glucuronidase activity assay following the reported procedures (Mordaka and Heap 2018).

### Fermentation

The modified PETC medium (Leang et al. 2013) supplemented with 5 g/l of fructose as the sole carbon source was used for heterotrophic fermentation experiments. For autotrophic fermentation, 50 ml of PETC medium was added into 260-ml serum bottles, and two types of gas mixtures were used as carbon sources, namely PETC[CO/CO_2_] and PETC[CO_2_/H_2_] (“Bacterial strains and growth conditions” section) pressurized to 0.2 Mpa in headspace. All PETC medium were supplemented with 5 µg ml^−1^ clarithromycin for plasmid maintenance and 10 ng ml^−1^ aTc to induce dCas9 expression.

Then, mid-log phase cultures of the transformants well adapted in the PETC[Fructose], PETC[CO/CO_2_], or PETC[CO_2_/H_2_] were transferred into the medium containing the corresponding carbon resource with an initial OD_600nm_ of 0.01, respectively. Transformants carrying plasmids pMTL-Tet3n0-Control or pMTL-IPL12-Control were used as the control strain. All serum bottles were incubated overnight at 37 ℃ followed by shaking at 100 rpm for 5–7 days. The culture was sampled every 12 h (during the exponential phase) or 24 h.

### Product quantification

To quantify the fermentation products, *C. ljungdahlii* cultures were centrifuged, and 1-ml supernatant was filtered through a 0.22-µm syringe filter into high-performance liquid chromatography (HPLC) vials. Fermentation products were analyzed using an Agilent 1200 HPLC, equipped with RID 1 A refractive index detector and HPX-87H 300 mm × 7.8 mm column. The column was maintained at 40 °C using slightly acidified water (5 mM H_2_SO_4_) as the mobile phase at a flow rate of 0.6 ml min^−1^. A 10-µl sample volume was injected. Pure compounds were used to generate standard curves for the identification and quantification of the products. To ensure an accurate comparison of metabolite titers (g/l) among all strains with varying growth rates, the metabolite titers were also normalized to biomass by dividing by OD_600_, yielding values in g·l^−1^·OD_600_^−1^.

### Quantitative real-time PCR and assessment of polar effects

Mid-exponential phase cultures of *C. ljungdahlii* CRISPRi strains were obtained and stored at −80 ℃ for total RNA isolation. The E.N.Z.A Bacterial RNA Kit was used to extract the total RNA according to the manufacturer’s instructions. Then, the concentration of extracted RNA was assessed by a Nano Drop spectrophotometer, and the quality was estimated using gel electrophoresis analysis. The resulting RNA samples were stored at −80 ℃. Then, HiScript III RT SuperMix for qPCR (+gDNA wiper) (Vazyme, China) was used to synthesize cDNA from RNA samples after genomic DNA removal. qPCR was performed with ChamQ SYBR Color qPCR Master Mix (Vazyme Biotech Co., Ltd., Nanjing, China) according to the manufacturer’s guidelines on a Roche LightCycler 480 Real-time PCR System (Roche, Switzerland). Reactions for three biological replicates of each group were carried out in 96-well plates. The housekeeping gene *rho* (CLJU_c02220, encoding transcription termination factor) was used as the internal standard (Zhao et al. 2019). Primers used for qPCR analysis in CRISPRi strains are listed in Table S2.

To evaluate possible polar effects, the operon structures associated with each target gene were analyzed. In addition, mRNA levels were quantified for co-transcribed genes within the same operon, which were positioned downstream of the targets. Altered expression in these neighboring genes was considered indicative of a potential polar effect.

### Cell morphology observation by microscopy

Phase-contrast microscopy was used to investigate cell morphology of *ftsZ* and *mre* CRISPRi strains. For samples preparation, these strains were cultured in PETC[Fructose] media with clarithromycin (5 µg/ml) and induced with 25 ng/ml aTc, and the cells were collected at the mid-log stage. Observations were performed using Zeiss Axio Imager.Z2 which was equipped with a 100 × oil immersion objective lens and phase contrast optics. Images were captured with Axiocam 208 color and analyzed by using ZEN 3.5 (Blue edition) software.

## Results

### Construction of an all-in-one plasmid system for CRISPR interference in *C. ljungdahlii*

In the gas-fermenting model species *C. ljungdahlii*, one fine system capable of tuning gene expression across a broad range is necessary for elucidating gene functions and regulating carbon flux toward desired products, which can ensure the sufficient knockdown magnitude and avoid excessive downregulation of essential genes potentially leading to cell death. The CRISPRi system can provide a good tool to finely regulate gene expression, and has been designed for those applications (Woolston et al. 2018).

When constructing an inducible CRISPRi system, an inducible promoter controlling the expression of the dCas protein is necessary. Two aTc-inducible promoters (Tet3n0 and IPL12) have been reported in *C. autoethanogenum*, which is able to regulate the expression of the chloramphenicol acetyltransferase gene ranging from 1 to 10.8 and 10 to 390 folds, respectively (Nagaraju et al. 2016), and before that, the dose-dependent inducibility of similar aTc-inducible promoters was demonstrated by GusA reporter activity (Dong et al., 2012). Here, with *gusA* as the reporter gene, the strength of these two promoters was assayed in *C. ljungdahlii*. The results showed that, without aTc, there is some leaky expression of *gusA* for both IPL12 and Tet3n0 (Fig. 1C). Upon induction with 35 ng/ml aTc, which is a moderate concentration compared with previously reported values of 10 ng/ml (Woolston et al. 2018), 32 ng/ml (Nagaraju et al. 2016), and 100 ng/ml (Dong et al., 2012), Tet3n0 and IPL12 can enhance the gene expression by 10 folds and 23 folds, which show a similar trend compared to previous reports. 

The *dcas9* was cloned under the control of the inducible promoter IPL12 and Tet3n0 in the plasmid pMTL82254 (Heap et al. 2009), carrying an erythromycin resistance gene (*ermB*) (Fig. 1A). The strong constitutive promoter (P_*thl*_) was used to drive the expression of sgRNA (Huang et al. 2016). The all-in-one plasmid was named as pMTLdCas9-targetGene-gRNA. In order to test the efficiency of the CRISPRi system, three genes for ethanol production were selected including bifunctional aldehyde dehydrogenase (*adhE1)* and aldehyde:ferredoxin oxidoreductases (*aor1* and *aor2*). At least two gRNAs targeting each gene were designed, respectively. One gRNA, in combination with either the IPL12 or Tet3n0 promoter driving dCas9 expression, was selected for subsequent experiments. Selection criteria required that the gRNA-promoter pair supported strain viability in PETC medium while eliciting distinct growth phenotype differences compared to the control. When multiple gRNAs or promoters yielded similar phenotype differences, one was chosen at random. All the transformants were cultured in the PETC[Fructose] condition with clarithromycin for maintaining the CRISPRi plasmid. The expression of each gene was assayed by qRT-PCR, which showed that the expression levels of *adhE1*, *aor1*,* and aor2* were repressed by 0.31-, 0.47-, and 0.15-fold after the expression of *dcas9* induced by aTc (Fig. 1D). These results showed that this CRISPRi system is able to effectively repress the expression of target genes.

On the other hand, several control strains were constructed and assessed. Strains expressing dCas9 alone (without a gRNA cassette) were cultured both in the presence and absence of aTc induction. Additional controls included strains with plasmids carrying only a gRNA cassette or co-expressing dCas9 with a non-targeting gRNA. In all cases, no significant differences in growth rates were observed between induced and uninduced conditions. These results demonstrate that expression of dCas9 alone, gRNA alone, or dCas9 paired with a non-targeting gRNA does not repress gene expression and that aTc supplementation itself imposes no appreciable growth defect (Figs. S1 and 2). Besides, growth curves were compared between induced and uninduced cultures of the CRISPRi knockdown strains (Fig. S3). These analyses revealed minimal leaky expression from the system, with negligible impacts on the phenotype.

### Validation of CRISPRi system

**Fig. 2:**
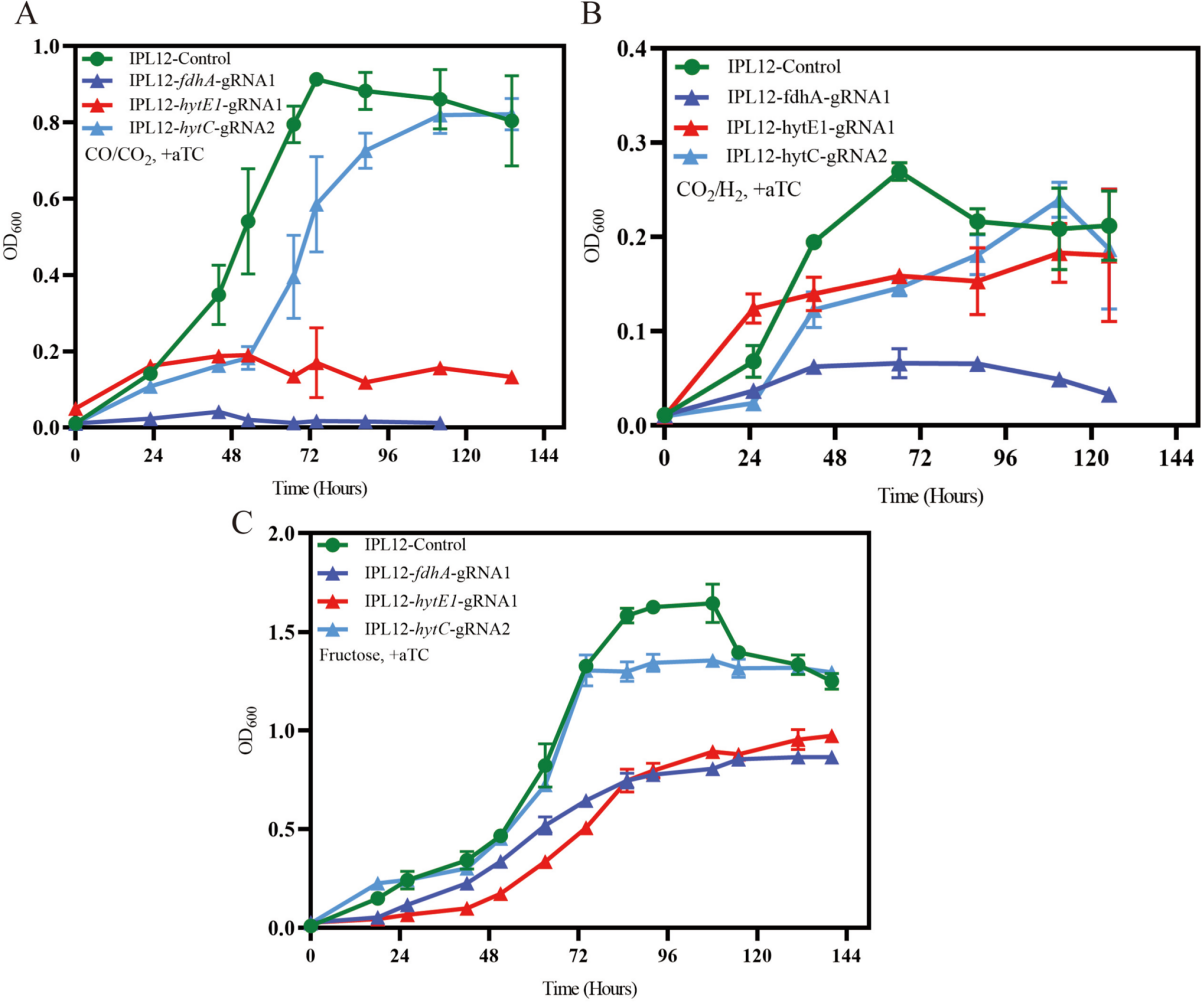
Growth changes of *fdhA*, *hytE1*, and *hytC* repressed strains caused by aTc-induced CRISPRi, compared to the control strain harboring pMTL-IPL12-Control, under **A** autotrophic conditions of PETC[CO/CO_2_] and **B** PETC[CO_2_/H_2_], and **C** the heterotrophic condition PETC[Fructose]. Data are representative of three replicates

In *C. ljungdahlii*, the Wood*-*Ljungdahl pathway is essential for the autotrophic metabolism, which is capable of converting the CO_2_/CO or CO_2_/H_2_ into acetyl-CoA as the precursor for the synthesis of main products (acetate and ethanol) and biomass. As proof of concept, three genes in the WL pathway were selected to validate the application of the CRISPRi system. For each gene, two unique gRNAs were designed. The CLJU_c20040 encoding one formate dehydrogenase (FdhA), CLJU_c07060 encoding 4Fe-4S ferredoxin oxidoreductase (HytC) as one essential component in the bifurcating hydrogenase complex, and CLJU_c07030 encoding NADH dehydrogenase (HytE) were selected. As expected, the results of qRT-PCR revealed that the CRISPRi system reduced transcription of the targeted genes, with *fdhA*, *hytC*, and *hytE* exhibiting 0.5-fold, 0.32-fold, and 0.45-fold decreases in mRNA levels, respectively (Fig. 1D). Consistent with these reductions, the repressed strains displayed severe growth impairment compared to controls when cultivated under PETC[CO/CO_2_], PETC[CO_2_/H_2_], or PETC[Fructose] conditions (Fig. 2). Of them, the *hytC* CRISPRi strain showed a similar growth curve as the control under three culture conditions, which may indicate that there are other genes with similar function in the genome, which can complement the expression regression of HytC under CO_2_/H_2_. These results are as expected and showed that the CRISPRi system can be considered one reliable method used for the study on the function of essential genes in *C. ljungdahlii*.

### The application of CRISPRi system in the carbon anabolic metabolism pathway

**Fig. 3:**
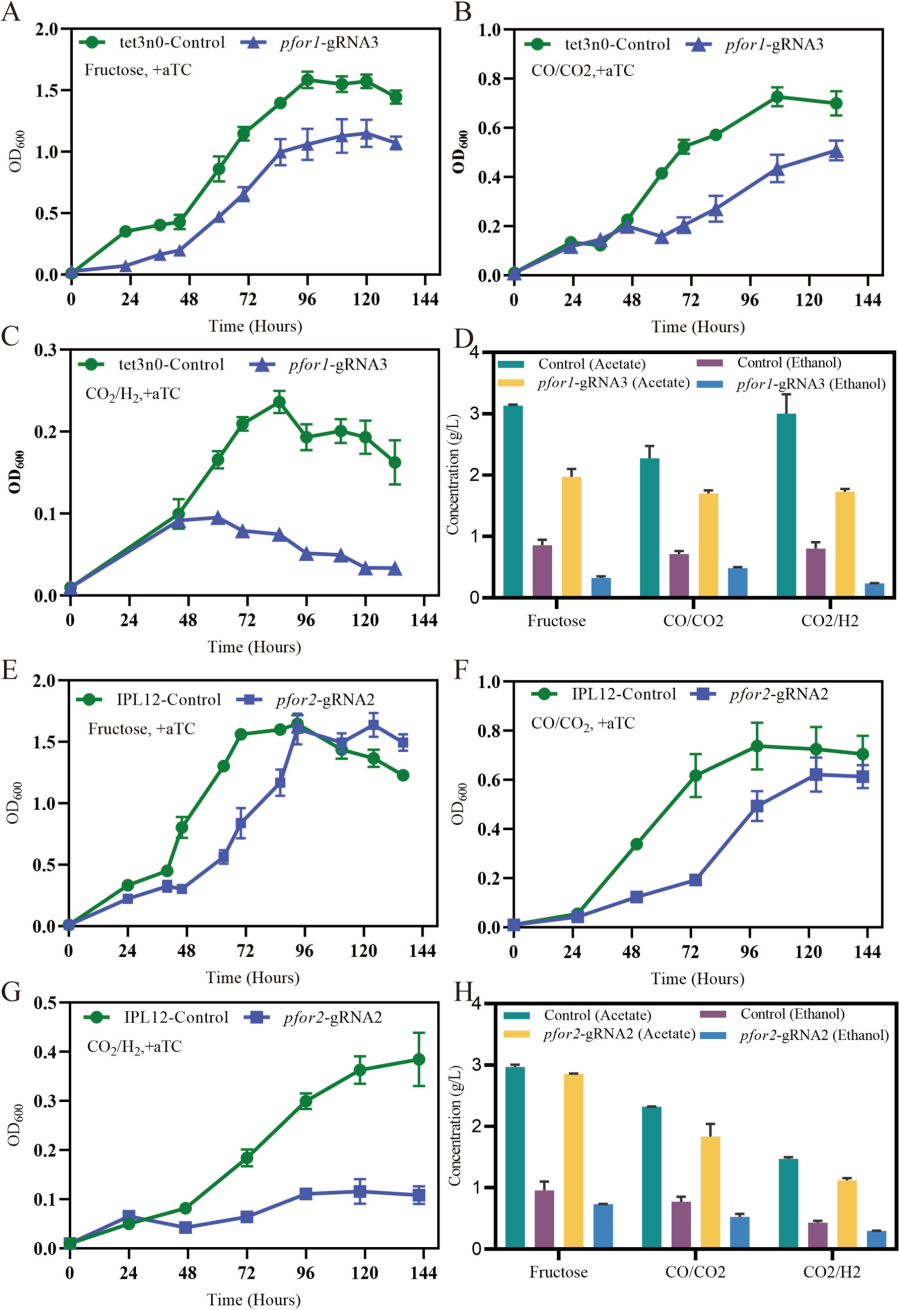
Phenotypic effects of *pfor1* or *pfor2* repression in *C. ljungdahlii* CRISPRi strains. **A**–**C**, **E**–**G** Growth changes of *pfor1* or *pfor2* repressed strains under autotrophic conditions (PETC[CO/CO_2_] and PETC[CO_2_/H_2_]), and the heterotrophic condition PETC[Fructose]. **D**, **H** Product (acetate and ethanol) concentrations of *pfor1* or *pfor2* strains under different conditions, compared to the control. All strains were cultured in triplicates

Until now, there are several toolboxes developed for the genetic manipulation of *C. ljungdahlii* (Huang et al. 2016; Leang et al. 2013), and CRISPRi provided a rapid tool for the study of the function of essential genes (Müh et al. 2019). Pyruvate:ferredoxin oxireductase catalyzed the conversion of acetyl-CoA to pyruvate, which is a critical step for biomass formation under autotrophic growth (Köpke et al. 2010; Nagarajan et al. 2013). There are two genes encoding pyruvate:ferredoxin oxireductase, and the expression of these two genes is different under gas; *pfor1* (CLJU_c09340) is expressed more highly (1000 folds) than *pfor2* (CLJU_c29340) (Al-Bassam et al. 2018; Tan et al. 2013; Zhu et al. 2020). Until now, the functions of the two genes are not known. Which gene is more important for autotrophic growth? Two unique gRNAs were designed for targeting the two genes, respectively. After the expression of *pfor1* was repressed, the mutant grew more poorly than the control under three conditions, especially under gas conditions, together with acetate and ethanol significantly decreased. By contrast, after the expression of *pfor2* was repressed, the growth of the mutant was less inhibited under both fructose and gas. When metabolite data were normalized to biomass (g·l^−1^·OD_600_^−1^) to eliminate effects arising from differences in growth rate or biomass yield, mutant strains normally exhibited elevated acetate yields alongside reduced ethanol production under some gas conditions (Fig. 6). These findings indicate that both genes support bacterial proliferation under heterotrophic and autotrophic conditions, with *pfor1* playing a particularly critical role during syngas-dependent (autotrophic) growth (Fig. 3). These phenotypes likely stem from their involvement in the reversible conversion of acetyl-CoA to pyruvate. Moreover, as PFOR relies on ferredoxin as a cofactor, repression of these genes disrupts intracellular redox balance, thereby altering carbon flux partitioning between acetate and ethanol.

**Fig. 4:**
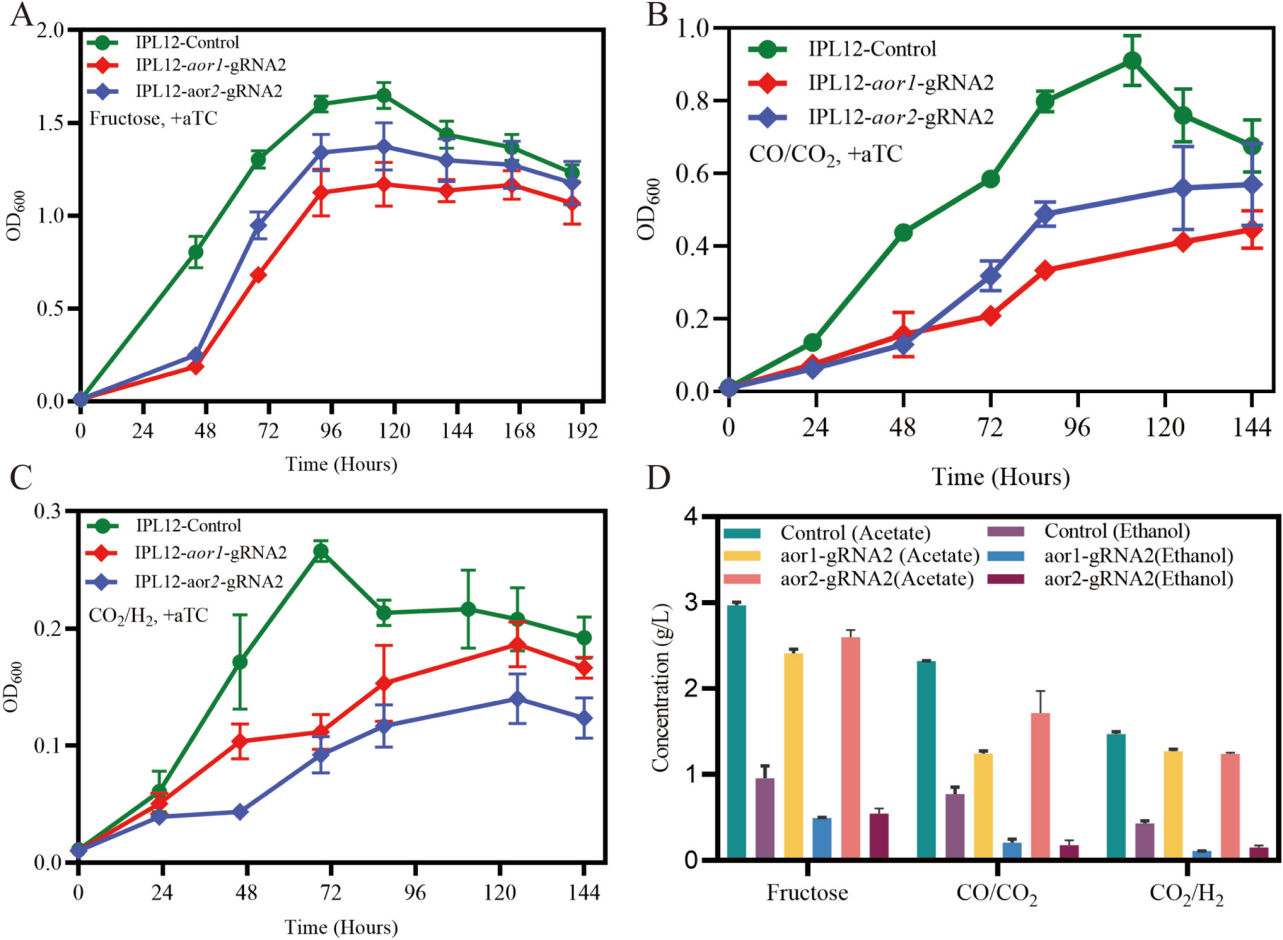
Growth curves (**A**–**C**) and product concentrations (**D**) of *aor1* or *aor2* CRISPRi strains of *C. ljungdahlii* grown under heterotrophic and autotrophic conditions. All mutants were cultured in triplicates

Acetaldehyde:ferredoxin oxidoreductase (AOR) catalyzes the reversible interconversion of acetate and acetaldehyde using ferredoxin as the electron donor or acceptor, and plays a crucial role in alcohol production (Liew et al. 2017). In *C. ljungdahlii*, it has been reported that *aor2* exhibits a significantly higher expression level than *aor1* under both autotrophic and heterotrophic conditions (Al-Bassam et al. 2018; Nagarajan et al. 2013; Zhu et al. 2020). However, their roles in autotrophic and heterotrophic metabolism remain incompletely characterized. In this study, CRISPRi targeting *aor1* or *aor2* was performed respectively to knock down their expression and investigate the resulting phenotypes. The results demonstrated that the two resulting strains exhibited significant growth inhibition under both autotrophic and heterotrophic conditions. Furthermore, the downregulation of *aor1* or *aor2* led to a marked decrease in titers of both acetate and ethanol (Fig. 4). However, it is noteworthy that both strains showed higher fractional molar yield (FMY) of acetate and corresponding lower FMY of ethanol in the fermentation products (Table S3). When normalized to biomass, acetate production remained generally elevated, while ethanol production was reduced compared to the control under autotrophic conditions (Fig. 6). These findings underscore the critical roles of both *aor1* (despite its natively low expression) and *aor2* in regulating carbon flux distribution during product synthesis.

**Fig. 5:**
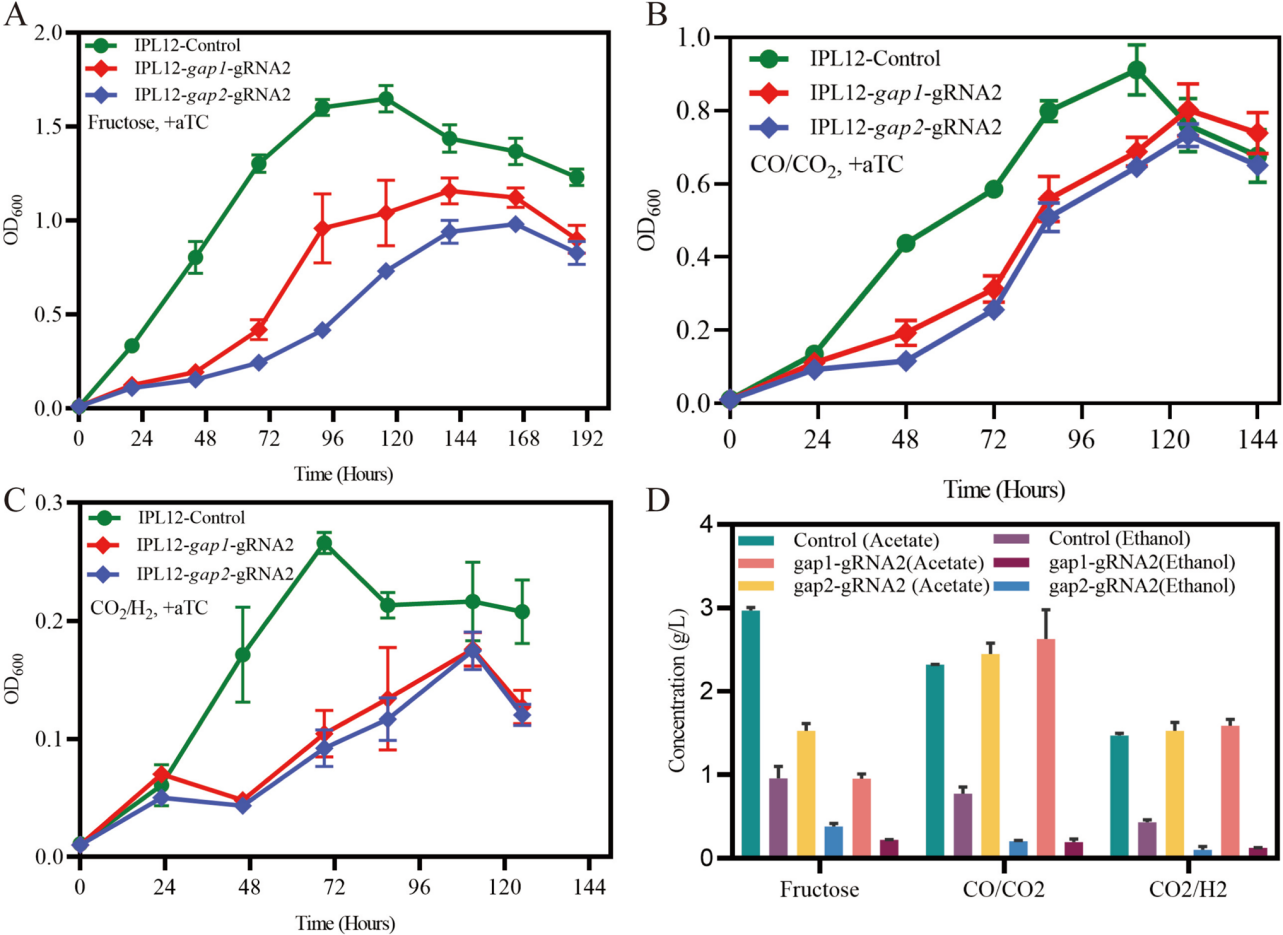
Growth curves (**A**–**C**) and product concentrations (**D**) of *gap1* or *gap2* CRISPRi strains of *C. ljungdahlii* grown under heterotrophic and autotrophic conditions. All mutants were cultured in triplicates

**Fig. 6:**
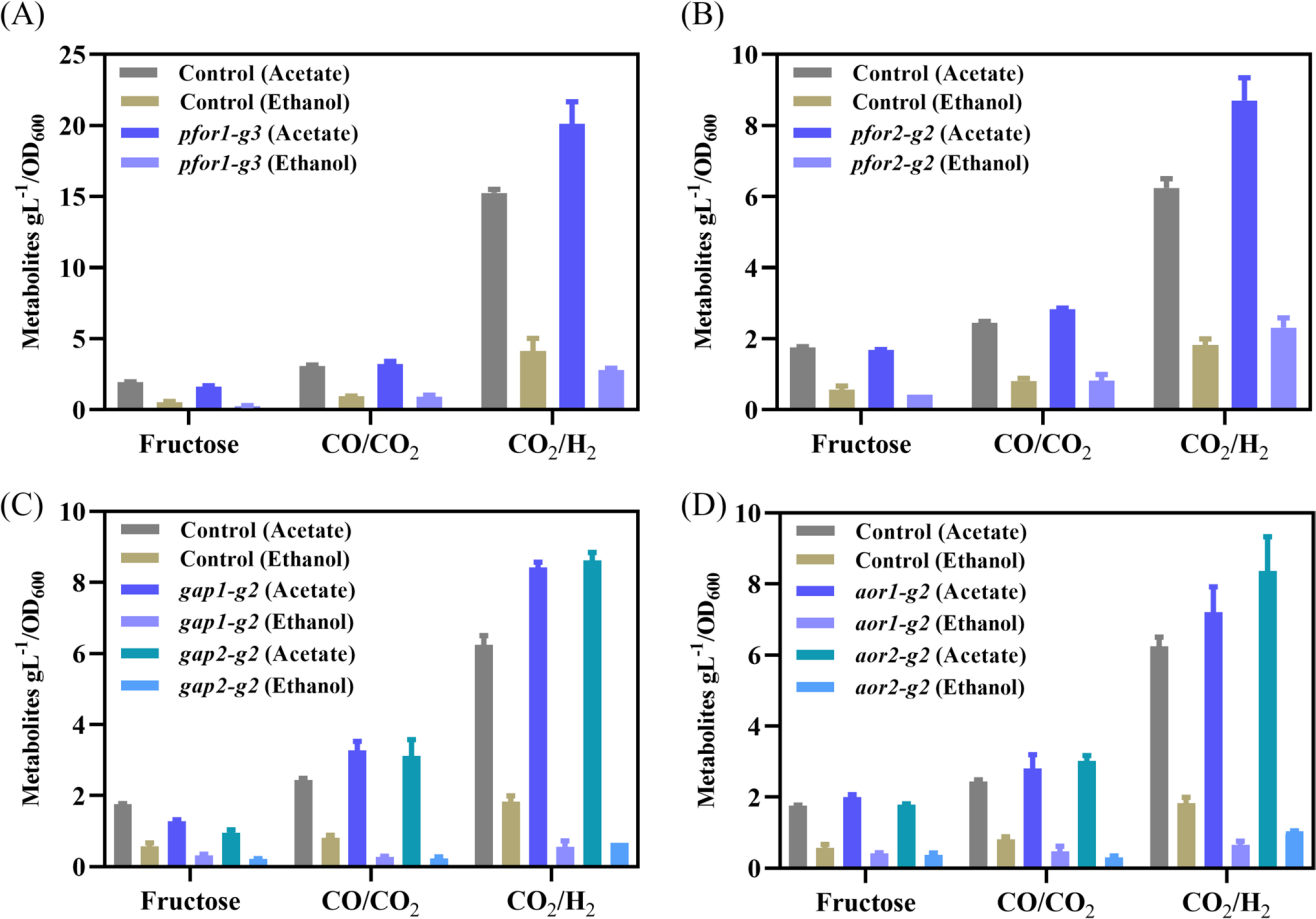
Normalized metabolite concentrations in CRISPRi knockdown strains. **A** *pfor1* knockdown, **B** *pfor2* knockdown, **C** *gap1* and *gap2* knockdowns, and **D** *aor1* and *aor2* knockdowns. All strains were cultured in triplicate

Glyceraldehyde-3-P is one important intermediate from the syngas to C6 and C5 compounds such as fructose and ribulose in the process of gluconeogenesis. In the *C. ljungdahlii* genome, there are two genes *gap1* and *gap2* (CLJU_c13400 and CLJU_c39150) encoding the glyceraldehyde-3-phosphate (G3P) dehydrogenase, which are critical genes for the conversion of 1,3-biphosphoglycerate into glyceraldehyde-3-P. Under CO_2_/CO or CO_2_/H_2_, these two genes are highly expressed and their expression is comparable; by contrast, the expression of CLJU_c39150 is about 4 folds greater than CLJU_c13400. Until now, the functions of the two genes are not known. Which gene is more important for autotrophic growth? Here, the expression of these two genes is repressed by the CRISPRi, and the phenotypes of the mutants are analyzed. The results showed that the growth of both *gap1* and *gap2* CRISPRi strains was inhibited under fructose, and the former strain grew much more poorly than the latter one. The products of acetate and ethanol were both decreased for the mutants. When culturing these two mutants under CO_2_/CO or CO_2_/H_2_, they showed similar growth curve and grew more poorly than the control strains. The products of these mutants were also analyzed; the production of acetate is comparable to the control strains. For the ethanol product, the mutants nearly do not produce ethanol, but the production of ethanol can achieve at 1 g/l for the control strain (Fig. 5). When normalized to OD_600_, knockdown strains exhibited elevated acetate titers and reduced ethanol titers compared to the control (Fig. 6). These findings demonstrate that *gap1* and *gap2* play comparable and essential roles in supporting the growth of *Clostridium ljungdahlii* under both autotrophic and heterotrophic conditions. The pronounced impairment in autotrophic growth highlights the critical involvement of GAPDH in gluconeogenesis, where it facilitates the synthesis of biomass precursors from acetyl-CoA. Moreover, the shift toward higher acetate and lower ethanol production per unit biomass implies that GAPDH contributes to intracellular NADH regeneration; its repression limits NADH availability, thereby redirecting carbon flux preferentially toward acetate formation.

### Morphology control via CIRSPRi

The genes *ftsZ* and *mreB* encode proteins responsible for the bacterial fission ring and cytoskeletal structures, respectively. Both of the genes *ftsZ* and *mreB* are essential for cell growth. These genes play a pivotal role in maintaining bacterial morphology, with *ftsZ* primarily regulating cell length and *mreB* controlling cell width (Elhadi et al. 2016). To investigate the roles of *ftsZ* (CLJU_RS03460) and *mreB* (CLJU_RS03475) in *C. ljungdahlii*, we used CRISPR interference (CRISPRi) to precisely repress their expression. CRISPRi can reduce gene expression without completely knocking out the gene. By examining the impact of gene repression on cell morphology, we aim to elucidate the functions of *ftsZ* and *mreB* in *C. ljungdahlii*.

**Fig. 7:**
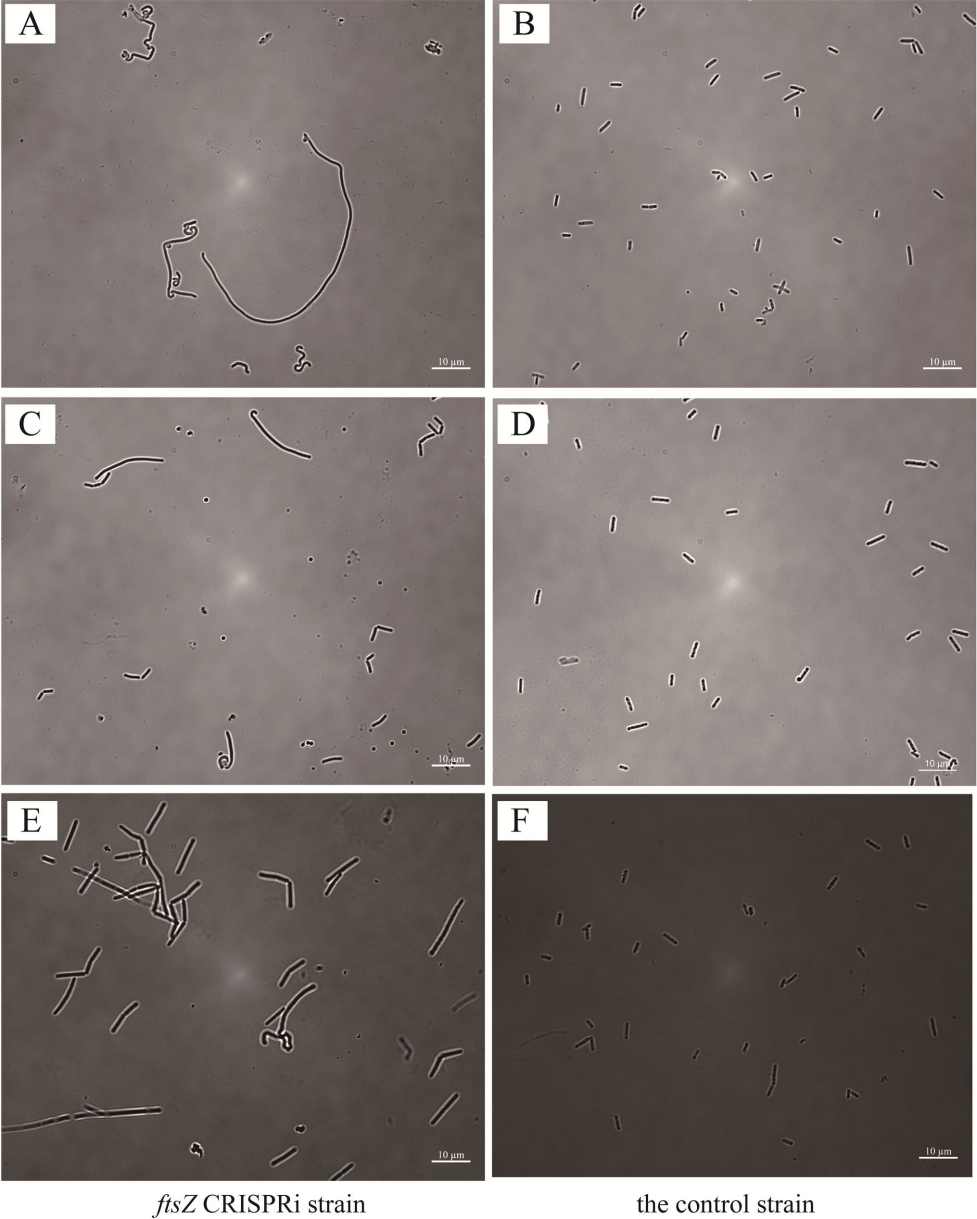
Microscopy studies on cell morphology of *C. ljungdahlii* strains harboring the CRISPRi plasmid targeting the *ftsZ* gene involved in cell division, compared to the control strain. Cells were grown in PETC[Fructose] (**A**, **B**), PETC[CO/CO_2_] (**C**, **D**), and PETC[CO_2_/H_2_] (**E**, **F**)

Our study underscored the critical roles of *ftsZ* in regulating cell division and morphology in *C. ljungdahlii*. The cell was elongated and the filamentous phenotype is observed in *ftsZ* mutant under fructose and gas conditions, compared to the control (Fig. 7), similar to previous reports in other bacteria like *E. coli* (Elhadi et al. 2016; Silvis et al. 2021) and *B. subtilis* (Koo et al. 2024; Peters et al. 2016), in which *ftsZ* proved to be fundamental to cell division. Our results extend these findings in *Clostridium* species, confirming that *ftsZ* regulates cell division in *C. ljungdahlii*. Compared to the wild-type strain, the *mreB* CRISPRi strains exhibited a significant difference in cell diameter. As observed in Fig. S4, cell diameters of the *mreB* repressed strain were ~0.84 µm, and those of the control strain were 0.62 µm, indicating an approximately 35% increase. This morphological change is kind of similar to the findings in other rod-shaped bacteria like *E. coli* (Koo et al. 2024) and *B. subtilis* (Peters et al. 2016), where the repression of *mreB* expression leads to increased cell diameter, although the magnitude of the effect observed here was less pronounced.

### Assessment of polar effects in CRISPRi-mediated knockdown strains

To assess the efficacy of gene repression and its polar effects of the CRISPRi system on, firstly, qRT-PCR was conducted for all targeted genes. The inducible CRISPRi system effectively repressed essential genes such as *pfor1*, *pfor2*, *gap1*, and *gap2*, leading to significant downregulation by 0.36-, 0.47-, 0.44-, and 0.45-fold, respectively (Fig. 1D). Similarly, the repression of cell morphology genes *ftsZ* and *mreB* was 0.61- and 0.54-fold, respectively. Regarding polar effects, operon analysis revealed that *aor1*, *aor2*, *pfor1*, *pfor2*, *gap1*, and *gap2* are monocistronic, and *ftsZ* was the terminal gene in its operon; thus, they were excluded from polar effect analysis. To examine polarity, transcription of downstream genes within selected operons was quantified, including CLJU_c20040–CLJU_c20030 (encoding *fdhA* and *hydN*), CLJU_c07030–CLJU_c07080 (encoding *hytCBDE*_*1*_*AE*_*2*_), and CLJU_c07190–CLJU_c07250 (containing *mreB*). The downstream genes *hydN* (CLJU_c20030), *hytB* (CLJU_c07040), *hytA* (CLJU_c07070), and *mreC* (CLJU_c07230) exhibited repression of 0.74-, 0.23-, 0.66-, and 0.20-fold, respectively (Fig. S5). These results demonstrate varying degrees of polar effects and confirm the capacity of the CRISPRi system to repress entire operons.

## Discussion

In this study, we developed an inducible CRISPR interference (CRISPRi) system that enables the precise and tunable regulation of essential genes in *C. ljungdahlii.* As the inducible promoter available for this species is limited, two aTc-inducible promoters from other species were applied to effectively tune the gene expression. With these two promoters driving the expression of the *dcas9* gene, we extended the genetic toolbox of *C. ljungdahlii* by developing an inducible CRISPRi system. Compared with the knockout of a gene, the system allowed rapid knockdown of gene expression just by replacing the gRNA expression cassette with the method of quick three-fragment ligation system developed in this study. The system has been demonstrated to effectively tune the expression of the genes involved in the essential gene for syngas utilization and carbon anabolism.

High-throughput functional genomics has emerged as a powerful tool for elucidating the roles of essential genes in growth under different carbon sources. For instance, Tn-seq-based methods have been successfully applied to investigate the genetic determinants of heterotrophic and autotrophic growth in *C. autoethanogenum* (Woods et al. 2022), a species closely related to *C. ljungdahlii*. Additionally, a constitutive promoter-driven CRISPRi system has been developed and utilized to study the function of essential regulators involved in autotrophic growth in *C. ljungdahlii* (Zhang et al. 2024). However, these methods are limited in studying temporal and conditional gene repression. The genome of *C. ljungdahlii* harbors numerous noncoding regulatory RNAs and unidentified regulatory sequences at a genome-wide scale. Our inducible CRISPRi system uniquely addresses this gap by enabling dynamic gene regulation and examination during specific growth phases or environmental conditions. Unlike traditional approaches, our system is especially valuable for autotrophic acetogens, whose gene essentiality depends on gaseous substrates and redox balance. By supporting the development of the partial genome-wide knockdown library, the system accelerates strain improvement and the systematic study of noncoding RNAs and regulatory sequences that affect CO_2_ fixation and product synthesis. These capabilities offer unique opportunities for innovative metabolite optimization and industrial applications, allowing applied scientists to use our tool effectively from the outset.

The efficacy of CRISPRi-mediated repression was confirmed by significant downregulation of target genes across all tested knockdown strains. Consistent with previous reports (Peters et al. 2016), CRISPRi targeting within polycistronic operons often exerted polar effects, leading to altered expression of adjacent genes. Notably, most knockdown strains exhibited repression of downstream genes, including *fdhA*, *hytC*, *hytE*, and *mreB*. The monocistronic genes *aor1*, *aor2*, *gap1*, and *gap2*, along with *ftsZ* (the terminal gene in its operon), were excluded from polar effect analysis. These observations carry important implications for interpreting phenotypic outcomes. Due to the prevalence of polar effects, any observed phenotypic changes in knockdown strains targeting operon-internal genes should be attributed potentially to simultaneous repression of multiple genes within the operon rather than solely to suppression of the intended single target gene, such as *fdhA*, *hytC*, *hytE*, and *mreB*. On the positive side, the robust polar repression underscores a key strength of the CRISPRi platform, namely its capacity to achieve simultaneous downregulation of multiple functionally related genes in a single operon. This feature represents a valuable expansion of the genetic toolkit for manipulating gene expression levels, enabling efficient probing of pathway-wide effects or coordinated knockdowns.

Knockdown of essential genes revealed that key metabolic enzymes control carbon and energy metabolism. In *Clostridium ljungdahlii*, PFOR catalyzes the synthesis of pyruvate from acetyl-CoA and CO_2_, serving as a critical node that connects the Wood-Ljungdahl pathway to central metabolism (Zhang et al. 2020). Knockdown of PFOR reduces pyruvate availability, thereby promoting acetyl-CoA accumulation for alternative product synthesis (e.g., acetate or ethanol) while minimizing carbon loss via decarboxylation. Similarly, suppression of glyceraldehyde-3-phosphate dehydrogenase (GAPDH) impedes the conversion of 1,3-bisphosphoglycerate (1,3-BPG) to glyceraldehyde-3-phosphate (G3P), which may redirect metabolic flux toward target pathways (Jia et al. 2021). However, the CRISPRi-mediated knockdown of PFOR and GAPDH in this study unexpectedly reduced acetate and ethanol production. This outcome likely stems from growth impairment in the engineered strains, underscoring the essential role of these enzymes in sustaining cellular viability under both heterotrophic and autotrophic conditions. More importantly, dynamic control of CRISPRi should be prioritized when engineering metabolic pathways involving viability-associated enzymes. Inducible knockdown strategies could be implemented once biomass accumulation reaches sufficient levels to withstand corresponding negative impacts to reroute carbon flux toward desired products.

In acetogens, there are two ethanol production routes: one route depends on the AdhE; the other is based on AOR, in which AOR catalyzes the reversible interconversion of acetate and acetaldehyde using ferredoxin as the electron donor or acceptor. The AOR-dependent route has been proposed to be the main route of ethanol production by several studies, allowing autotrophically producing ethanol while generating ATP (Mock et al. 2015). In *C. autoethanogenum* (closely related to *C. ljungdahlii*), the expression of *aor1* (CAETHG_0092) is significantly higher than that of *aor2* (CAETHG_0102) (Marcellin et al. 2016). Inactivation of either the *aor1* or *aor2* gene resulted in a prolonged growth lag phase and reduced the final cell density to approximately half of that of the wild type when CO was used as the carbon source. However, the final cell density of these strains remains unaffected during growth on fructose. In terms of product formation, the *aor1* knockout (KO) strain of *C. autoethanogenum* produced lower amounts of ethanol and 2,3-butanediol, while the acetate level remained unchanged during growth on both CO and fructose, suggesting that a higher proportion of carbon went into acetate. In contrast, in the *aor2* KO strain, the proportion of carbon directed to acetate decreased, while the percentage directed to ethanol correspondingly increased during growth on both CO and fructose. In *C. ljungdahlii*, AOR1 and AOR2 share 100% protein sequence identity with the corresponding proteins of *C. autoethanogenum* (Table S4). However, in contrast to *C. autoethanogenum*, *aor2* is expressed at a significantly higher level than *aor1* in *C. ljungdahlii* under both autotrophic and heterotrophic conditions (Al-Bassam et al. 2018; Liu et al. 2020; Nagarajan et al. 2013). In this study, we demonstrated that the partial downregulation of *aor1* or *aor2* resulted in impaired growth, reduced acetate and ethanol titers, and a notable increase in the fractional molar yield of acetate under one heterotrophic condition (PETC[Fructose]) and two autotrophic conditions (PETC[CO/CO_2_] and PETC[CO_2_/H_2_]), indicating that AOR1 and AOR2 primarily function in the reduction of acetate under these conditions, and their downregulation results in a higher proportion of carbon diverted to acetate. These findings indicate the role of AOR as an arbiter in determining acetate:ethanol ratios in *C. ljungdahlii*, as proposed by Richter et al. (2016). This highlights the distinct ethanol metabolism characteristics between *C. ljungdahlii* (Lo et al. 2020) and its close relative *C. autoethanogenum*, even though their genomes share more than 99% identity (Jones et al. 2016). This shows species-specific AOR regulation and distinct ways to balance carbon flux between acetate and ethanol production.

In addition to metabolic regulation, this study is the first to use the inducible CRISPRi system to investigate the function of two essential genes for cell division and morphology of *C. ljungdahlii*. Our results showed that repressed expression levels of *ftsZ* led to the formation of elongated cells, which showed a similar phenotype characterized in other bacteria (Elhadi et al. 2016), although the regulatory mechanism of these genes in *Clostridia* is inadequately known. Our finding provides valuable insights into their contributions to cell division and cell shape. Another gene *mreB* was reported to increase the diameter of cells after its expression is repressed (Peters et al. 2016). In this study, repression of *mreB* significantly increased cell diameter as expected (Fig. S4). Quantitative imaging was conducted to resolve the mechanistic contribution of *mreB* and its associated proteins in cell shape determination. The presence of a quantifiable phenotype confirms *mreB* repression and underscores the use of CRISPRi to analyze the cell-shape determining gene in *C. ljungdahlii.*

Our results demonstrate that inducible gene repression can reveal species-dependent functions of essential genes within the unique metabolic framework and regulatory features of acetogens. This underscores the need for a tailored genetic toolkit to unravel the regulatory mechanism in gas-fermenting bacteria.

## Conclusions

In this study, we developed an inducible CRISPRi system capable of wide-ranging regulation and validated its effectiveness by knocking down key genes in the Wood-Ljungdahl pathway of *C. ljungdahlii*. Furthermore, knockdown of PFOR and GAPDH-1 demonstrated the effectiveness of the CRISPRi system in key enzymes of central metabolism and inhibited cell viability, highlighting the importance of appropriate induction timing for inhibition to fully exploit the dynamic regulatory capacity of this system. Repression of the two *aor* genes revealed their roles in regulating acetate and ethanol production. Furthermore, downregulation of *ftsZ* underscored the feasibility of using the CRISPRi system to modulate the morphology of *C. ljungdahlii*, emphasizing the potential of this organism in morphological alterations. In conclusion, this study used the CRISPRi system to deepen our understanding of key gene functions, providing a valuable knowledge base and a powerful tool for metabolic engineering aimed at achieving desired phenotypes.

## Data Availability

The datasets generated during and/or analysed during the current study are available from the corresponding author on reasonable request.

## Information

Below is the link to the electronic supplementary material. ESM 1 (TIF 310 KB) ESM 2 (TIF 797 KB) ESM 3 (TIF 3.70 MB) ESM 4 (JPG 2.23 MB) ESM 5 (TIF 393 KB) ESM 6 (JPG 1.16 MB) ESM 7 (JPG 1.04 MB) ESM 8 (JPG 1.23 MB) ESM 9 (JPG 1.14 MB) ESM 10 (JPG 1.05 MB) ESM 11 (PNG 3.88 MB) ESM 12 (TIF 5.51 MB) ESM 13 (JPG 0.98 MB) ESM 14 (XLSX 10.8 KB) ESM 15 (XLSX 15.7 KB) ESM 16 (XLSX 16.2 KB) ESM 17 (XLSX 10.7 KB)
