# Supplementary figures and images for: Elucidating the roles of essential genes in autotrophic metabolism and cell morphology of Clostridium ljungdahlii by CRISPRi
Source: Appl Microbiol Biotechnol. 2026 Jan 27;110(1):44. doi: 10.1007/s00253-026-13714-3 (PMC12852162; doi:10.1007/s00253-026-13714-3)

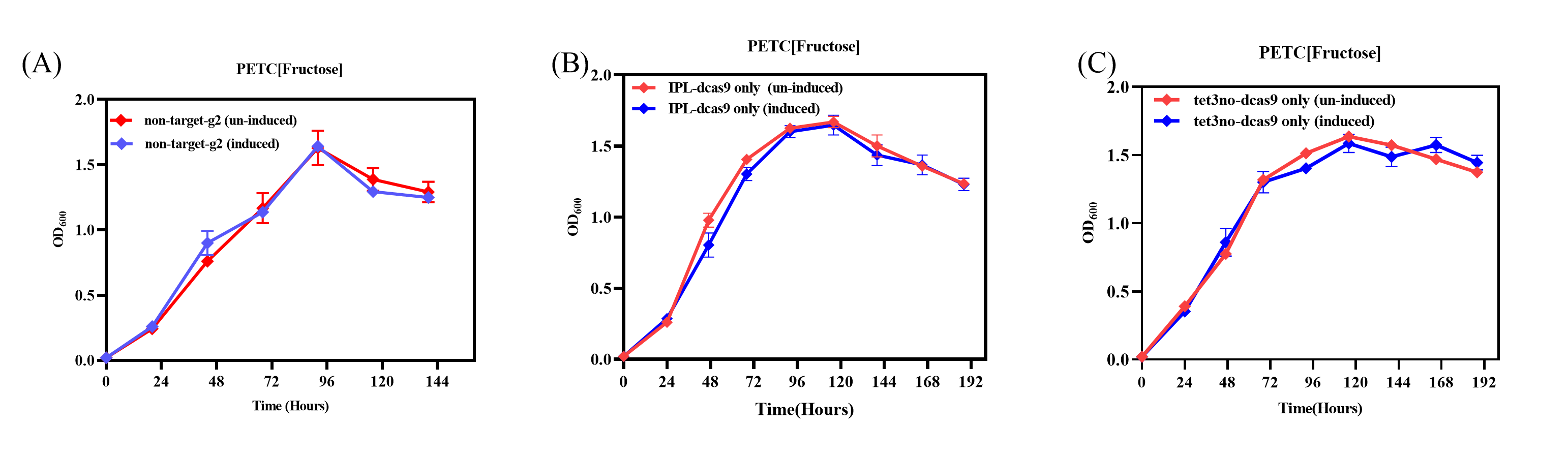

Supplement: ESM 1 — (TIF 310 KB) [file 253_2026_13714_MOESM1_ESM.tif]

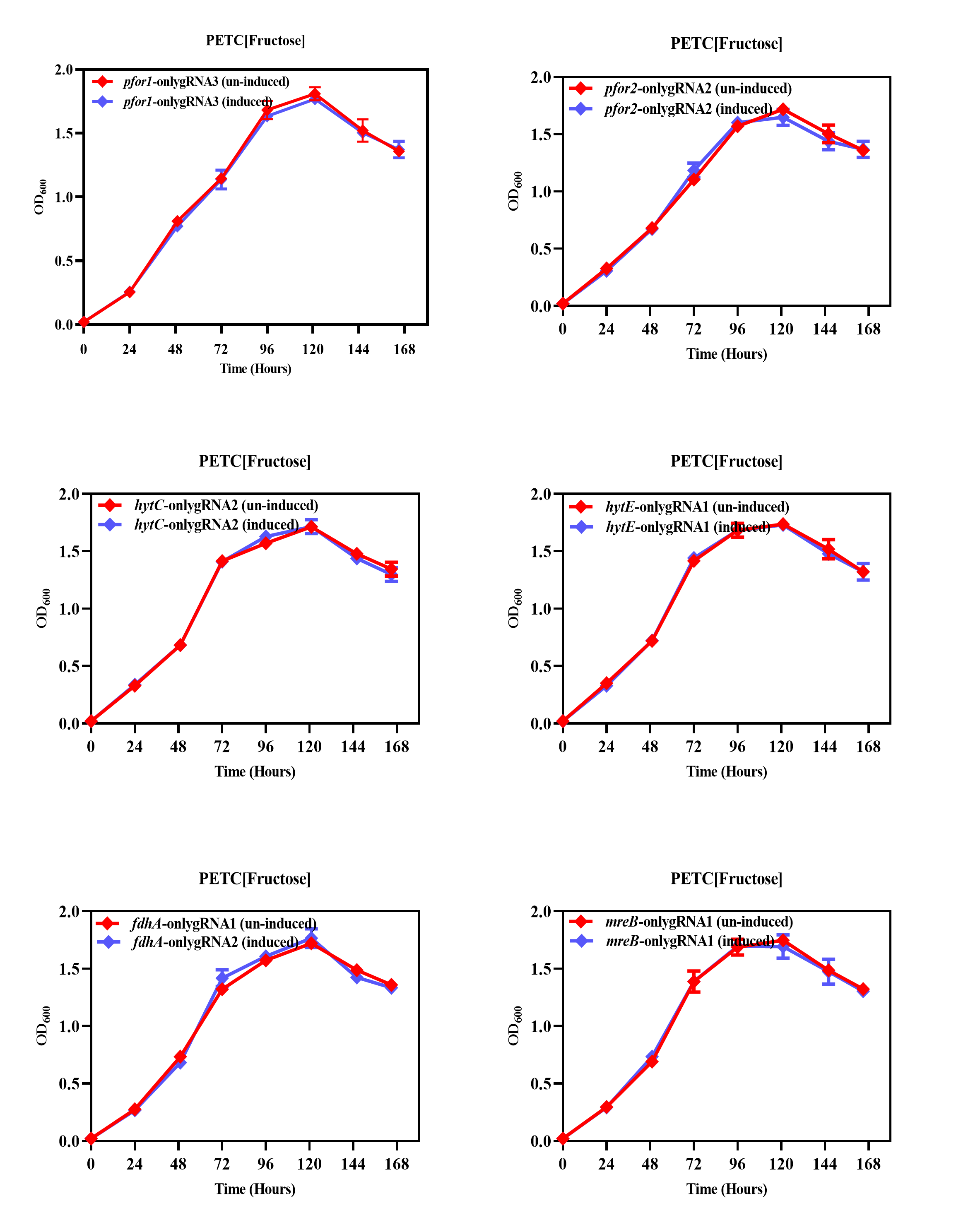

Supplement: ESM 2 — (TIF 797 KB) [file 253_2026_13714_MOESM2_ESM.tif]

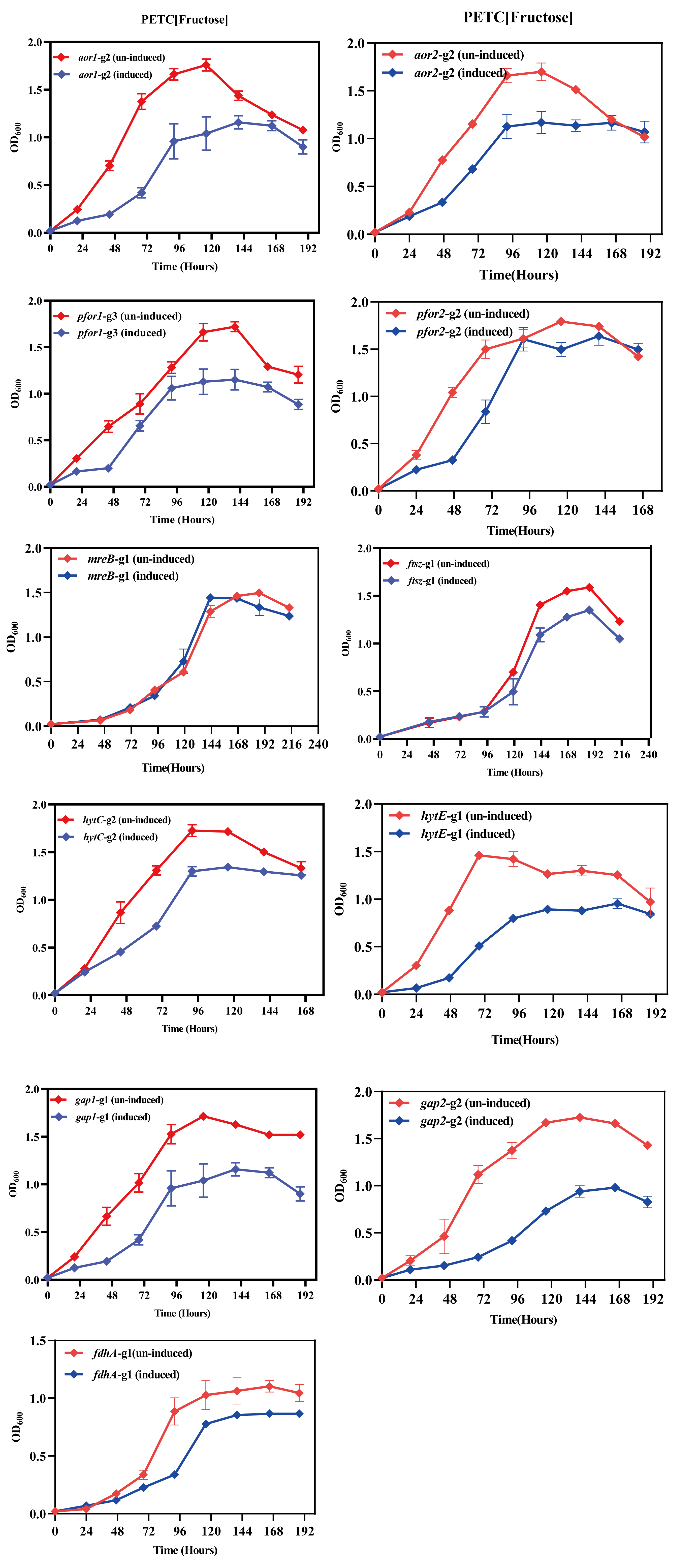

Supplement: ESM 3 — (TIF 3.70 MB) [file 253_2026_13714_MOESM3_ESM.tif]

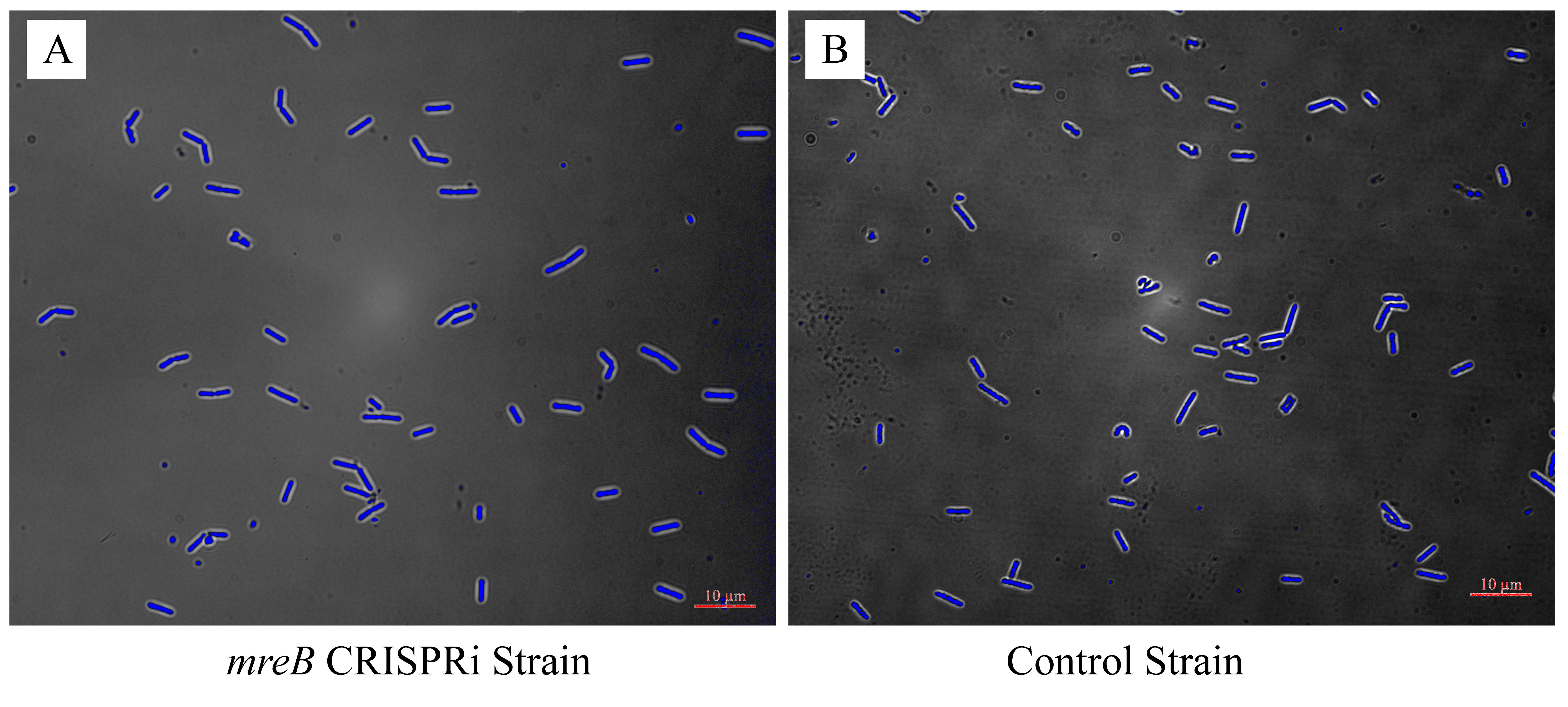

Supplement: ESM 4 — (JPG 2.23 MB) [file 253_2026_13714_MOESM4_ESM.jpg]

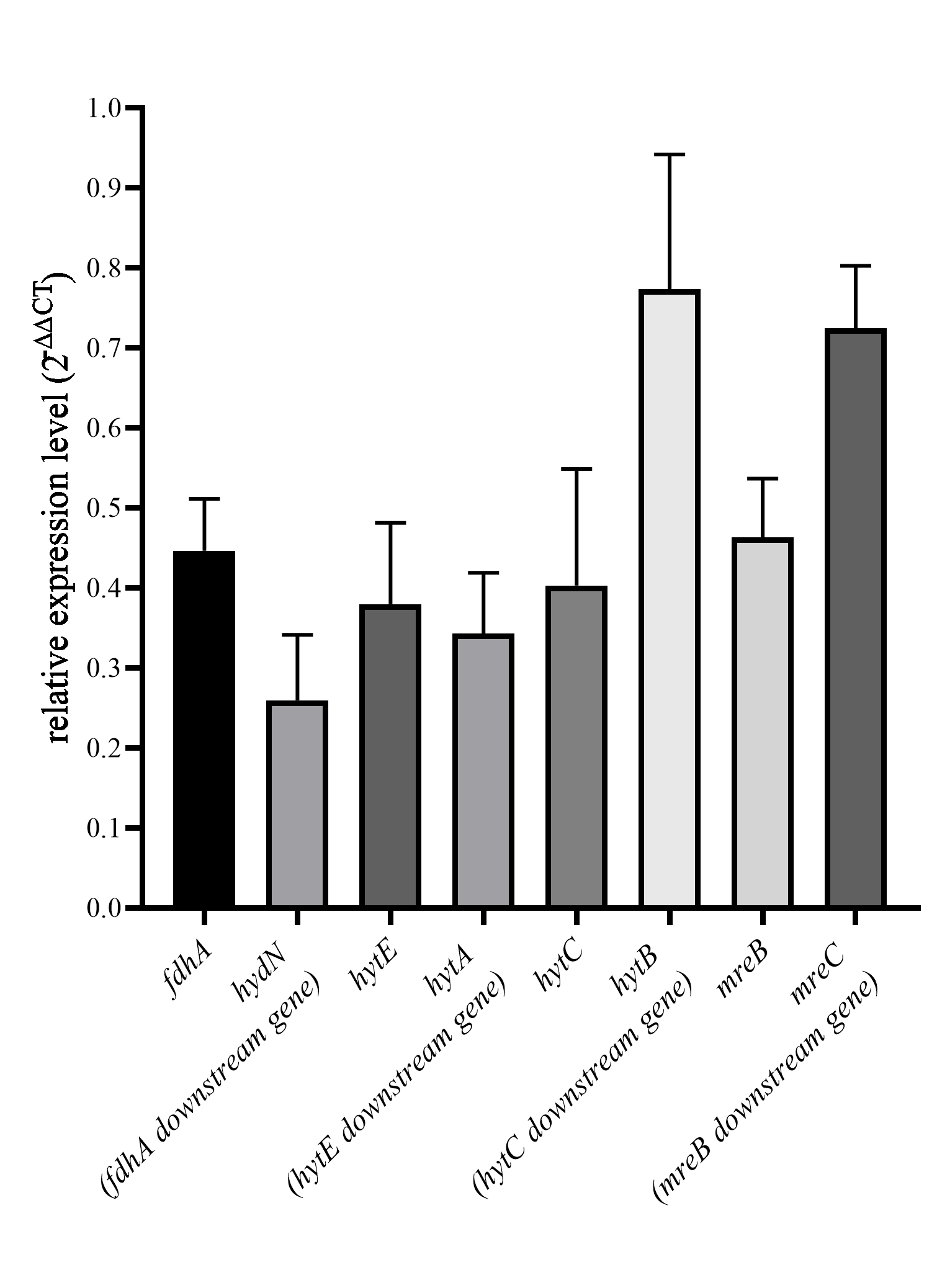

Supplement: ESM 5 — (TIF 393 KB) [file 253_2026_13714_MOESM5_ESM.tif]

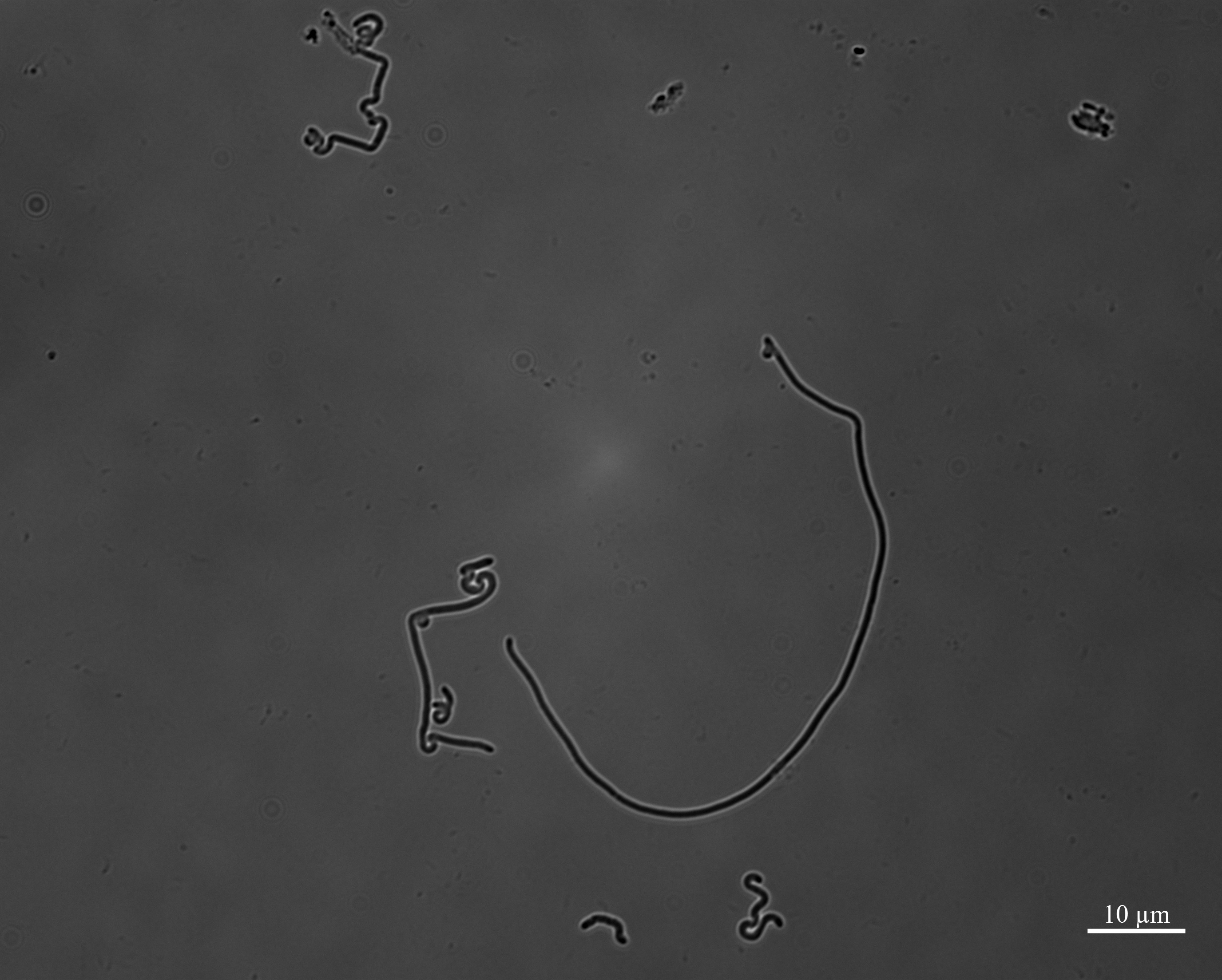

Supplement: ESM 6 — (JPG 1.16 MB) [file 253_2026_13714_MOESM6_ESM.jpg]

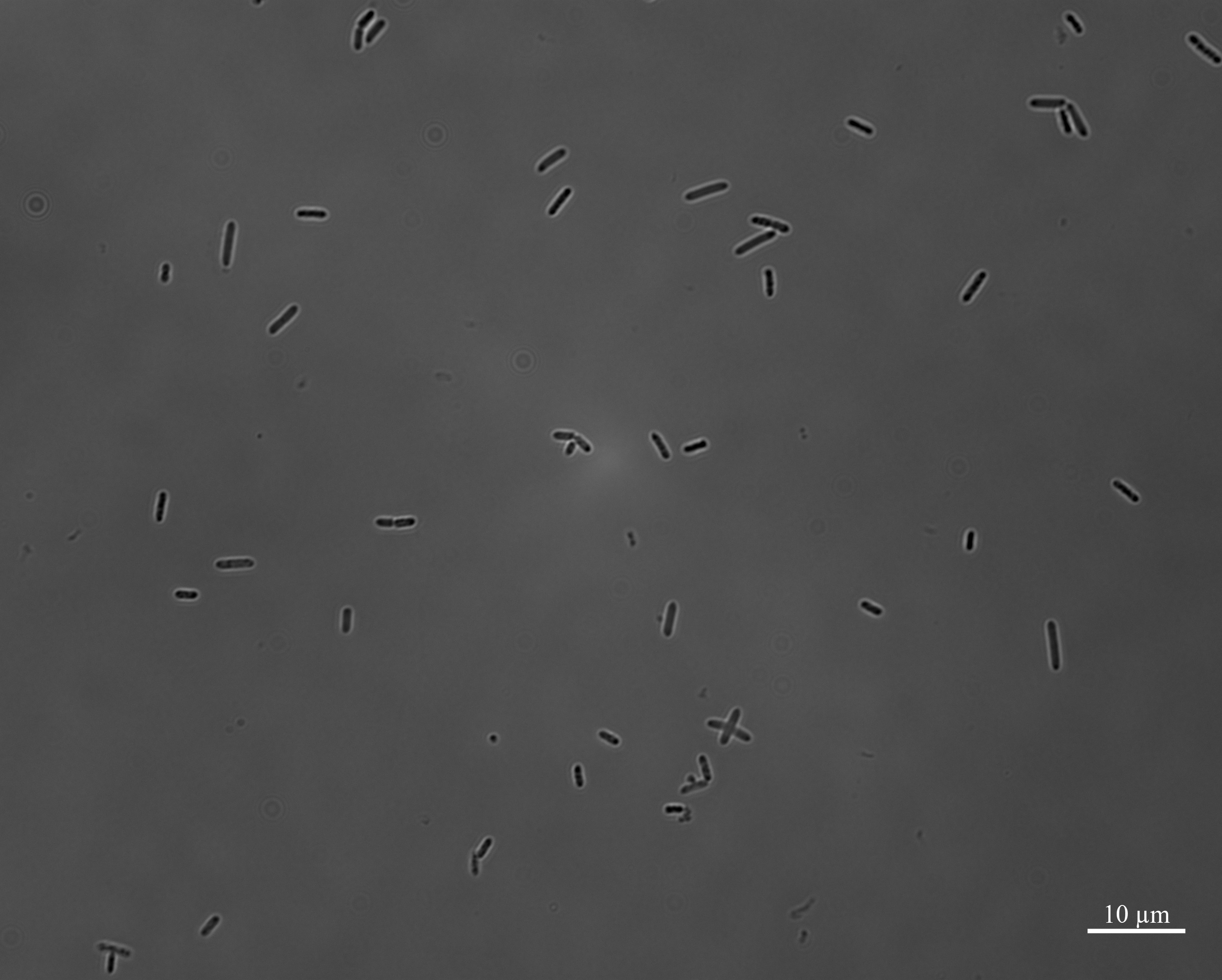

Supplement: ESM 7 — (JPG 1.04 MB) [file 253_2026_13714_MOESM7_ESM.jpg]

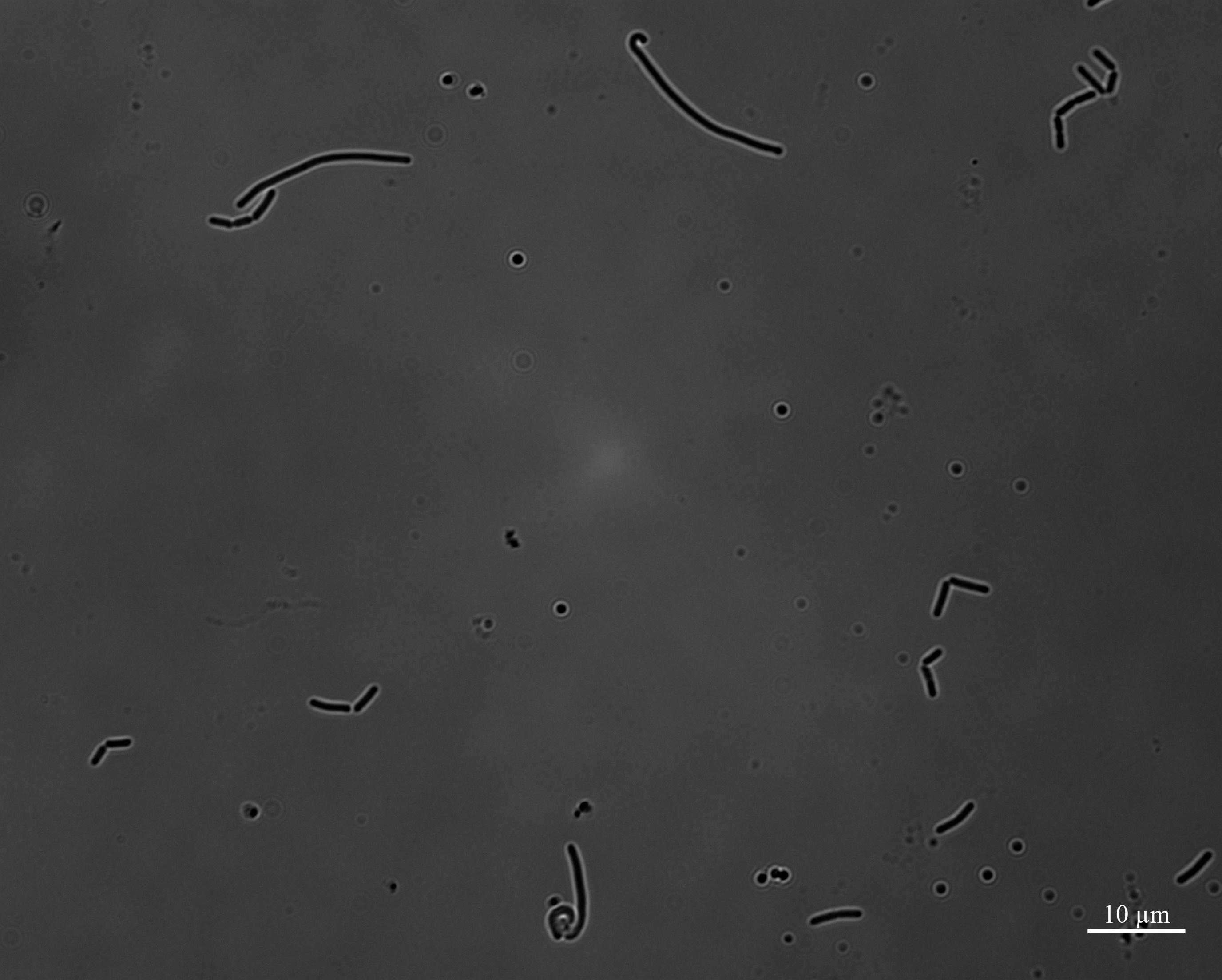

Supplement: ESM 8 — (JPG 1.23 MB) [file 253_2026_13714_MOESM8_ESM.jpg]

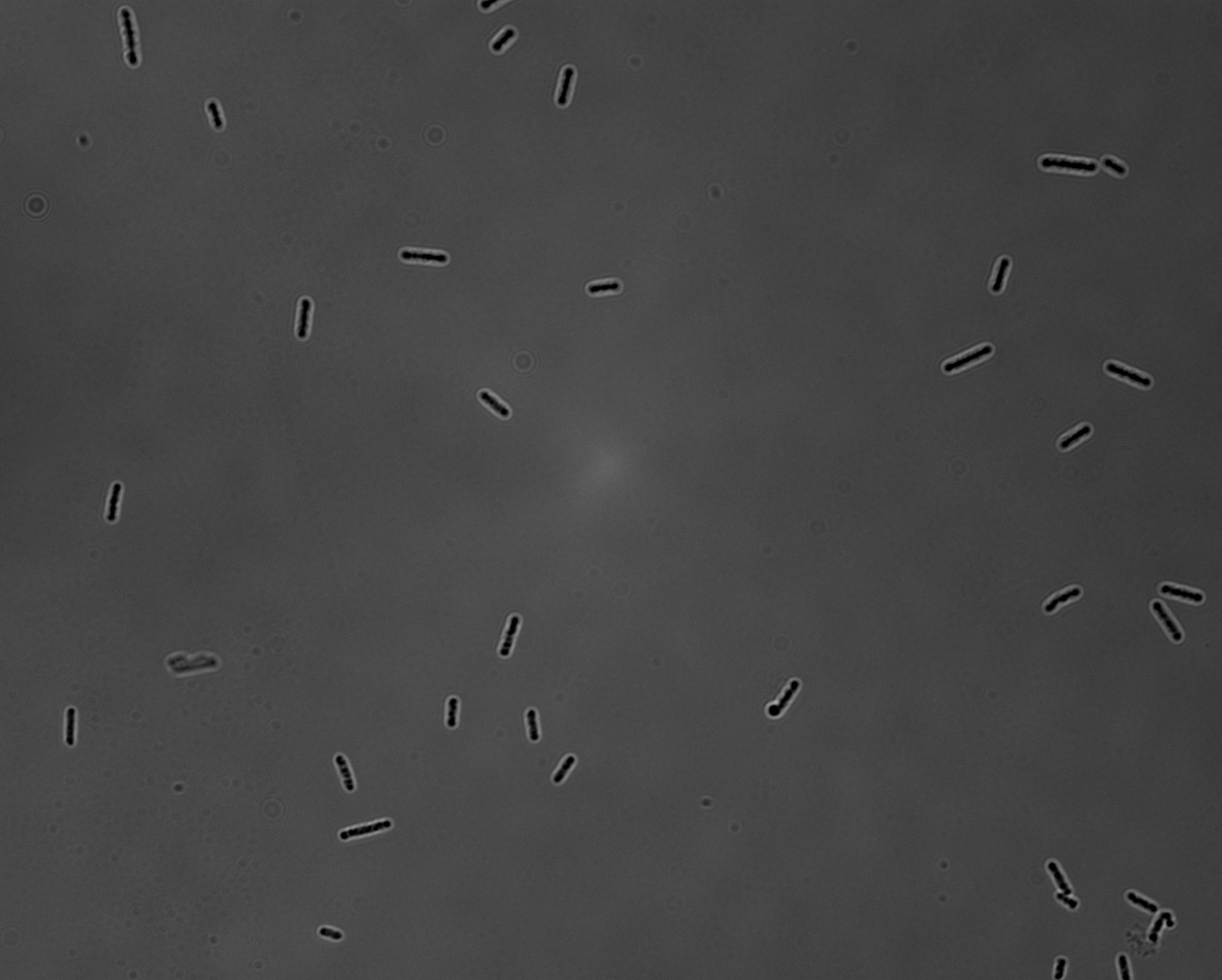

Supplement: ESM 9 — (JPG 1.14 MB) [file 253_2026_13714_MOESM9_ESM.jpg]

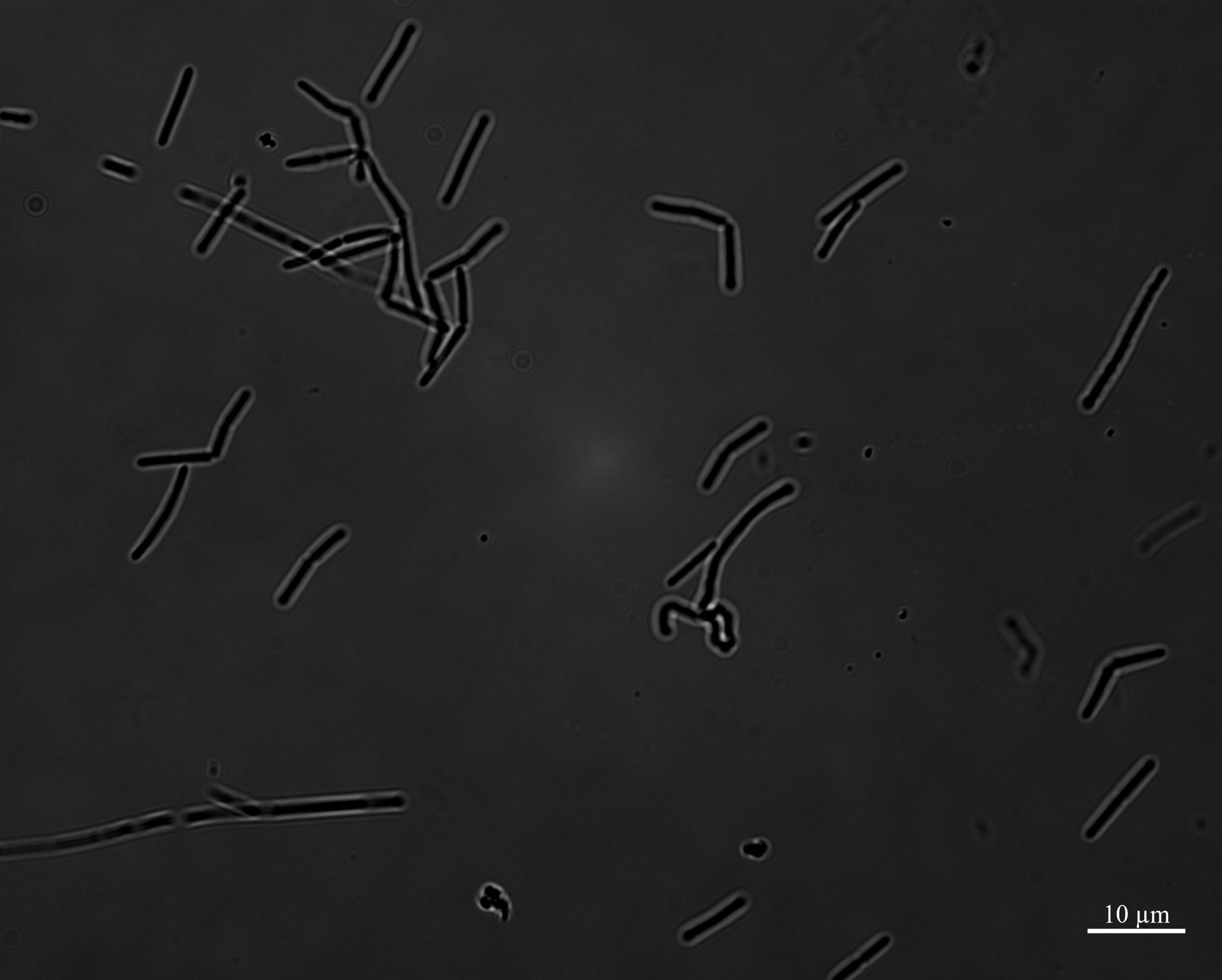

Supplement: ESM 10 — (JPG 1.05 MB) [file 253_2026_13714_MOESM10_ESM.jpg]

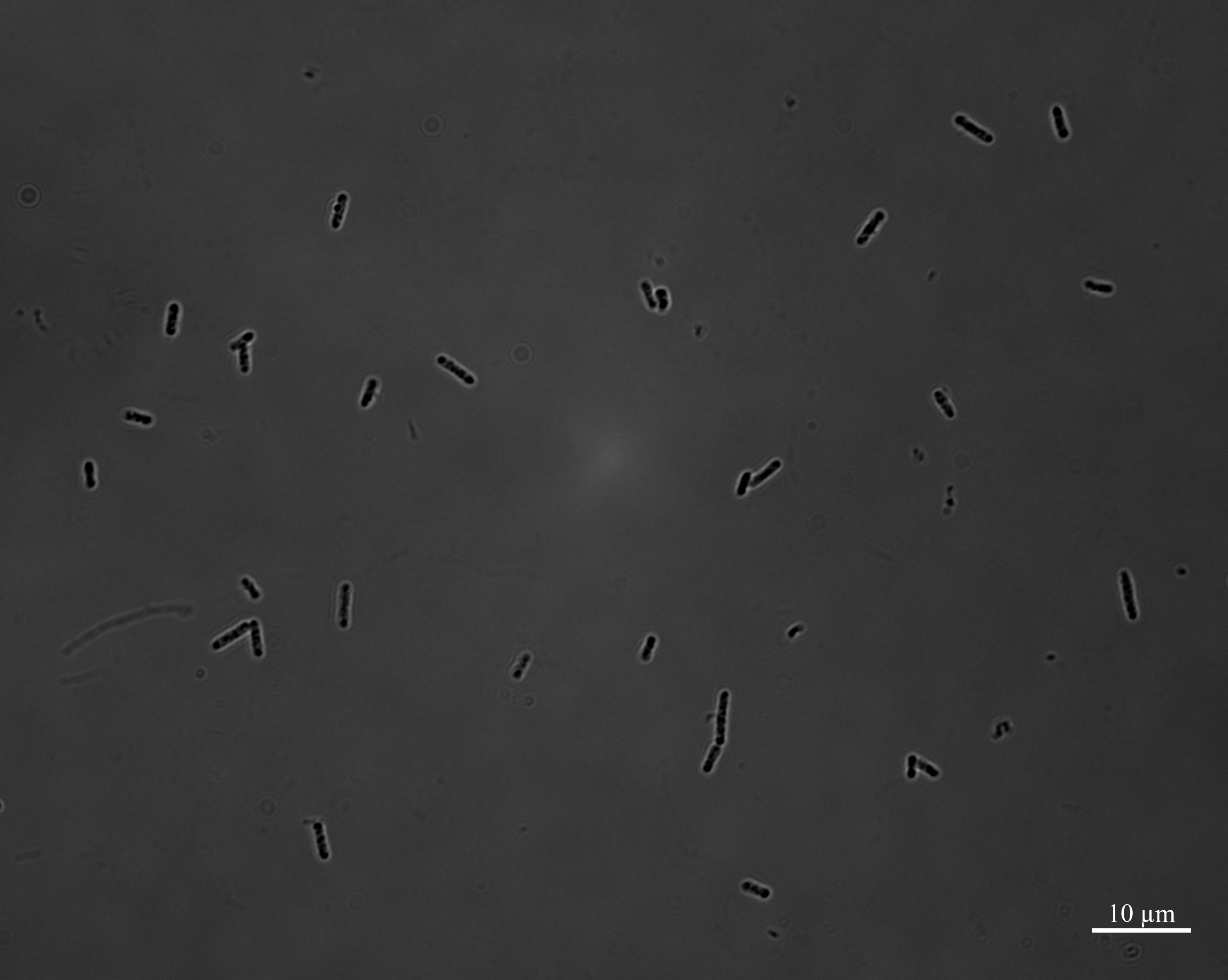

Supplement: ESM 11 — (PNG 3.88 MB) [file 253_2026_13714_MOESM11_ESM.png]

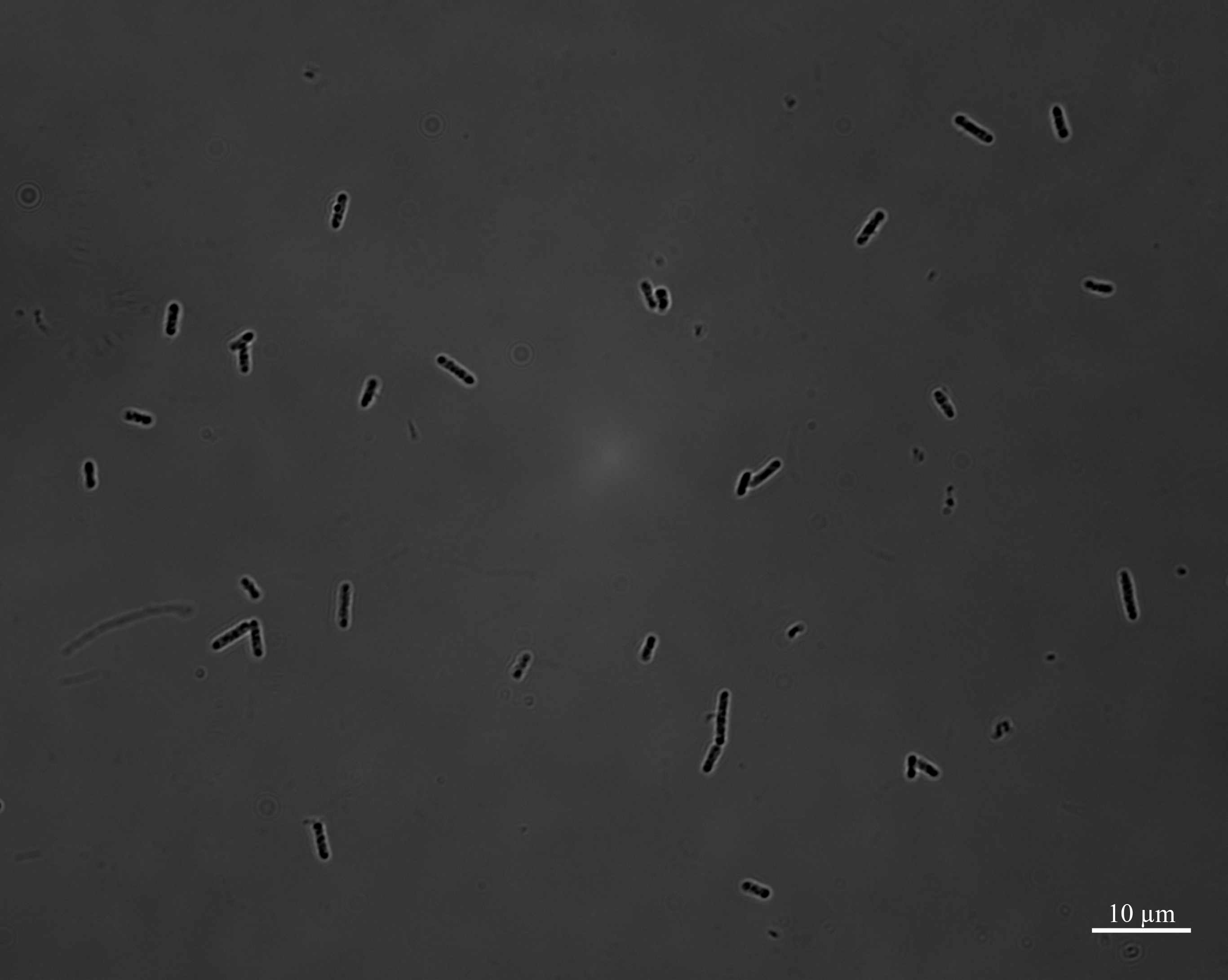

Supplement: ESM 12 — (TIF 5.51 MB) [file 253_2026_13714_MOESM12_ESM.tif]

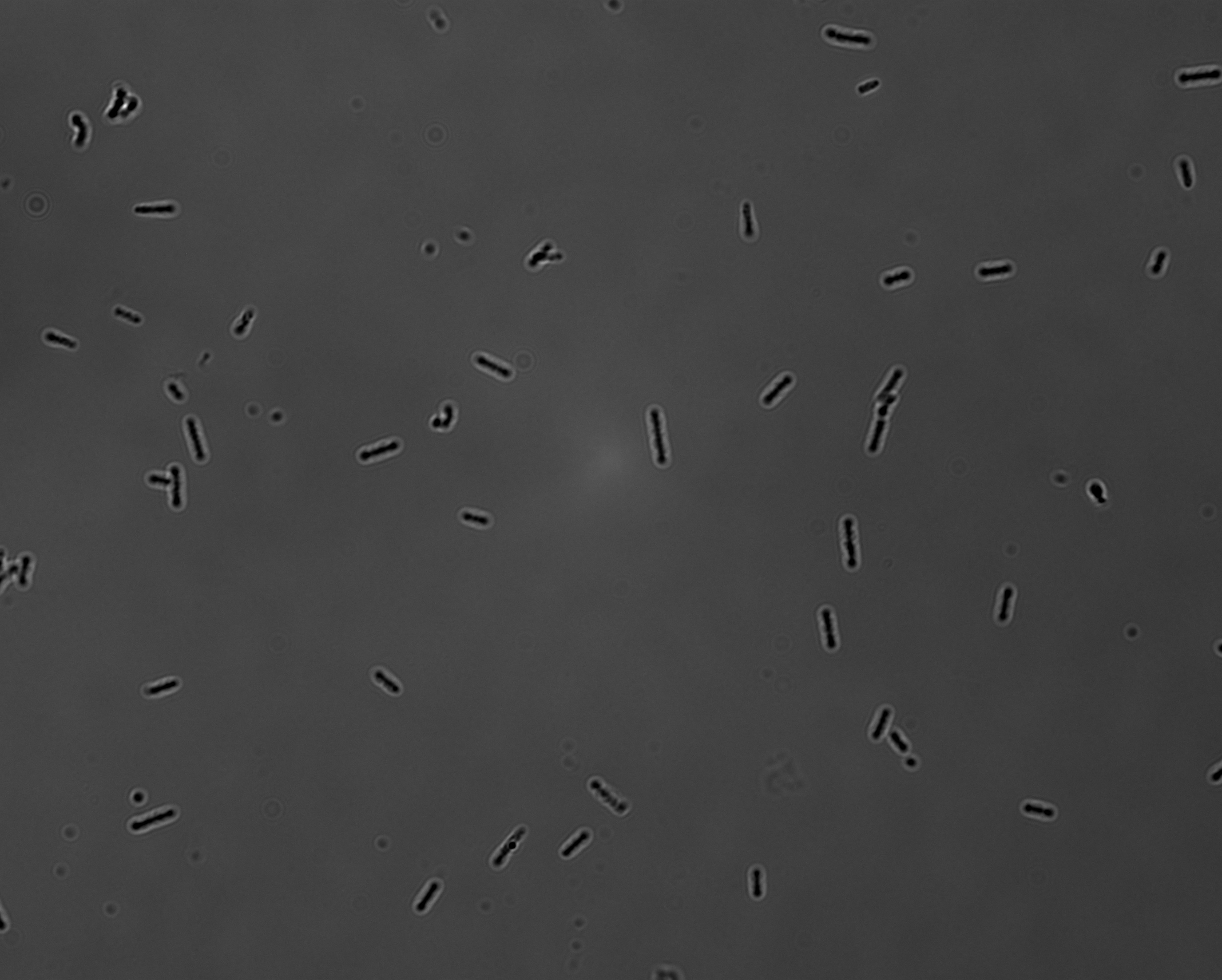

Supplement: ESM 13 — (JPG 0.98 MB) [file 253_2026_13714_MOESM13_ESM.jpg]
